# The Impact of Musculoskeletal Injuries Sustained in Road Traffic Crashes on Work-Related Outcomes: A Systematic Review

**DOI:** 10.3390/ijerph182111504

**Published:** 2021-11-01

**Authors:** Elise M. Gane, Melanie L. Plinsinga, Charlotte L. Brakenridge, Esther J. Smits, Tammy Aplin, Venerina Johnston

**Affiliations:** 1 School of Health and Rehabilitation Sciences, The University of Queensland, Brisbane 4072, Australia; t.aplin1@uq.edu.au (T.A.); v.johnston@uq.edu.au (V.J.); 2 Recover Injury Research Centre, The University of Queensland, Brisbane 4066, Australia; m.plinsinga@uq.edu.au (M.L.P.); c.brakenridge@uq.edu.au (C.L.B.); e.smits@uq.edu.au (E.J.S.); 3 Physiotherapy Department, Princess Alexandra Hospital, Brisbane 4102, Australia; 4 Centre for Functioning and Health Research, Metro South Health, Brisbane 4102, Australia; 5 Allied Health Research Collaborative, The Prince Charles Hospital, Brisbane 4032, Australia

**Keywords:** musculoskeletal injury, traffic accidents, occupational rehabilitation, return to work

## Abstract

Musculoskeletal injuries occur frequently after road traffic crashes (RTCs), and the effect on work participation is not fully understood. The primary aim of this review was to determine the impact of sustaining a musculoskeletal injury during an RTC on the rate of return to work (RTW), sick leave, and other work outcomes. The secondary aim was to determine factors associated with these work-related outcomes. An electronic search of relevant databases to identify observational studies related to work and employment, RTC, and musculoskeletal injuries was conducted. Where possible, outcome data were pooled by follow-up period to answer the primary aim. Fifty-three studies were included in this review, of which 28 were included in meta-analyses. The pooled rate of RTW was 70% at 1 month, 67% at 3 months, 76% at 6 months, 83% at 12 months, and 70% at 24 months. Twenty-seven percent of participants took some sick leave by one month follow-up, 13% by 3 months, 23% by 6 months, 36% by 12 months, and 22% by 24 months. Most of the factors identified as associated with work outcomes were health-related, with some evidence also for sociodemographic factors. While 70% of people with RTC-related musculoskeletal injury RTW shortly after accident, many still have not RTW two years later.

## 1. Introduction

Musculoskeletal injuries occur frequently after road traffic crashes (RTCs) to varying levels of severity. This includes whiplash injury, which is one of the most common injuries [1,2,3], along with milder injuries such as sprains and strains and more serious injuries such as fractures and dislocations. [4]. A range of negative health outcomes arise from musculoskeletal injuries, including disability and reduced health-related quality of life [5,6]. One understudied outcome of sustaining a musculoskeletal injury from an RTC is the impact on paid employment. The nature of the injury may require hospitalisation, which necessitates time off work. Even without hospitalisation, musculoskeletal injuries may leave individuals physically unable to work or with symptoms that interfere with work [7].

Work is important for many reasons, including the promotion of positive health outcomes. There is a recognized link between harm to mental and physical health, and prolonged time off work [8,9,10]. There is also a link between unemployment and increased mortality rates, as well as increased health service utilisation [11]. In addition, work is considered to be therapeutic—this is a founding principle of work disability management [12]. Musculoskeletal disorders is one of the top two diagnoses associated with long-term sick leave, the other being mental disorders [13]. Application of the biopsychosocial model in the successful rehabilitation of individuals with work-related disability is supported by evidence [14] as well as key regulatory authorities and state governments in Australia [15]. The psychological component of the biopsychosocial model is not to be ignored, as previous work has identified expectations of RTW to be significantly predictive of achieving RTW after injury or illness [16], including RTC specifically [17].

Recent work in the field of orthopaedic trauma (not specific to RTCs) suggests that 61% of injured persons RTW within 2 years post-injury [18] with an average of 100 days of work missed as a result [18]. Having such data regarding workers recovering from RTC-related musculoskeletal injuries would be helpful in assisting injured workers, their employers, and their health professionals understand the potential recovery trajectories and assist in setting rehabilitation and RTW goals. This information could also help to highlight the need for employer and legislated support with regard to RTW. In addition, more evidence is required for the factors associated with work and work-related outcomes in this cohort to inform workplace and employment support systems. A recent systematic review of personal and social factors associated with sustainable RTW (i.e., maintenance of RTW without sick leave) after musculoskeletal disorders and common mental disorders identified five key factors: support from supervisors and co-workers, a positive attitude, self-efficacy, younger age, and higher education levels [19]. Key injury-specific elements such as the influence of compulsory third-party schemes on work-related outcomes warrant further investigation in the post-RTC clinical population.

Delayed RTW and absence from work are not the only work-related outcomes that could be affected by an RTC-related musculoskeletal injury. Work capacity, work ability, and health-related work productivity loss are also key outcomes to consider. Work capacity is often considered to be an individual’s capacity to meet the demands of their job, and may require modification of duties or hours of work for example. Work capacity is an important construct when considering the outcomes of RTC, as medical clearance to RTW is often required either by the employer or the insurer [20]. Work ability is defined as the worker’s own perceptions of their health and their ability to meet the physical and mental demands of their job [21]. Previous research has shown that musculoskeletal injuries (not specific to RTC) affect workers’ occupational readiness and task performance [22], and are an independent predictor of impaired work ability [23]. Health-related work productivity loss is another important work outcome to consider as there are significant costs arising from lost productivity associated with musculoskeletal disorders—missed workdays due to low back pain in the US, for example, lead to US$28 billion in lost productivity annually [24]. Prior to designing interventions to promote RTW for those with musculoskeletal injuries after RTCs, more needs to be known about the nature of recovery for these individuals across a number of work outcome indicators.

The primary aim of this systematic review is to determine the impact of sustaining a musculoskeletal injury during an RTC on five work-related outcomes: the rate of RTW following injury, the utilisation of sick leave, work capacity, work ability, and health-related work productivity loss. The secondary aim is to determine factors associated with these work-related outcomes in people with musculoskeletal injuries as a result of an RTC.

## 2. Materials and Methods

This review was conducted and reported in compliance with the Preferred Reporting Items for Systematic Review and Meta-Analyses (PRISMA) [25]. This systematic review was registered in the International Prospective Register for Systematic Reviews (PROSPERO) on 14 August 2018 (reference number CRD42018099252). A detailed protocol on the methodological approach has been published [26].

### 2.1. Search Methods

An electronic search was conducted in PubMed, EMBASE, Cumulated Index to Nursing and Allied Health Literature (CINAHL), Web of Science, PsycINFO, and Australian Transport Index databases on 2 July 2020. To identify relevant articles, a combination of search terms was used related to work and employment, RTC, observational study design, and musculoskeletal injuries [26]. The search was limited to English studies and all publication years were considered. The search strategy was developed in PubMed and modified for the other databases. The PubMed search strategy can be found in Supplementary Material S1.

### 2.2. Eligibility Criteria

This systematic review included published studies of individuals who sustained musculoskeletal injuries during an RTC on work-related outcomes.

#### 2.2.1. Population

Studies were eligible for inclusion if participants were adults who sustained a musculoskeletal injury during an RTC. Studies that investigated outcomes after neurological injuries such as spinal cord injury and traumatic brain injury were excluded, as recent work has already been done in this space [27,28]. Other studies were excluded if the same participants had mixed musculoskeletal and neurological injuries (multisystem trauma). If the cohort was a mix of individuals with musculoskeletal injuries and individuals with neurological injuries, the study was included if results were presented separately for those with musculoskeletal injuries.

Adults were the focus of this review, and thus studies with exclusively paediatric cohorts were excluded. However, studies that consisted of a small percentage of adolescent participants were still included if adults remained the majority of the cohort [29,30,31,32]. This is a deviation from our published protocol [26]. The authors felt the inclusion of the highly relevant results from these studies added strength to this review, outweighed the disadvantage of deviating from the protocol, and recognized the fact that adolescents can be employed and experience a negative impact on their work if injured.

#### 2.2.2. Outcome

Five work-related outcomes were the focus of this review, and included studies needed to report at least one of: (i) RTW rate, (ii) sick leave, (iii) work ability, (iv) work capacity, or (v) health-related work productivity loss. These work-related outcomes could be reported as either a primary or secondary outcome of the study.

#### 2.2.3. Study Design

Included studies were observational in design, including cross-sectional, retrospective, and prospective. If participants engaged in rehabilitation programs that were considered ‘usual care’ or those that occur in ‘real world’ settings (e.g., participants would have received this treatment regardless of whether they were included in a research study or not), those studies were considered observational and were included in the present review. Studies were excluded if they were randomized controlled trials or case studies, or pre-post-intervention designs implemented by the study authors (not usual care). These criteria were applied in order to capture outcomes from participants recovering under ‘real world’ circumstances, and not experimentally controlled care.

#### 2.2.4. Language

Only studies published in English were eligible for this review.

### 2.3. Study Selection

Title and abstract screening and full-text screening was conducted independently by two researchers (divided among authors EG, ES, CB, TA) in Microsoft Excel. Author EG screened all title and abstracts, with the second screening conducted by TA (60%), CB (30%) and ES (10%), allocated at random. Full-text screening was equally distributed between EG, TA and ES. Allocation to author for screening occurred in a manner to enable equal crossover amongst the three authors (e.g., 50% of EG’s full texts were also reviewed by ES, and the other 50% by TA). Discrepancies were resolved by group discussion or by senior author VJ. Reference lists of included studies and relevant systematic reviews identified in the search were reviewed for eligible studies.

### 2.4. Data Extraction

Data extraction was divided evenly amongst the authors (EG, ES, CB, TA, MP) so that each study was examined independently by two authors, with consensus achieved by consultation with the senior author VJ. Allocation for data extraction was similar to full-text review in that there was approximately equal cross-over amongst authors. Data were extracted into a predetermined Excel form that has been described in detail previously [26]. Authors of included studies were contacted by authors EG or MP if data was incomplete or if data needed to be confirmed.

### 2.5. Quality Appraisal

Study quality was assessed with the National Institutes of Health (NIH) National Heart, Lung, and Blood Institute (NHLBI) Study Quality Assessment Tools for observational cohort and cross-sectional studies, and case–control studies [33]. Co-authors (EG, ES, CB, TA, MP) conducted two independent quality assessments of each result on the same articles on which they conducted data extraction and consensus was reached with the senior author VJ.

### 2.6. Analysis

Inter-rater reliability of the quality assessment tool was examined using kappa (κ) statistics (SPSS version 25.0; IBM Corp, Chicago IL). Reliability was considered as slight (0.00–0.2), fair (0.21–0.4), moderate (0.41–0.6), substantial (0.61–0.8) or almost perfect (0.81–1.0) [34].

Meta-analysis was conducted provided that at least two studies reported on the same work outcome including variability estimates. Categorical outcomes (e.g., percentage of people returned to work) were transformed using the Freeman-Tukey double arcsine method [35] and pooled prevalence scores were calculated in Medcalc (MedCalc Software bv Ostend, Belgium). No continuous data were identified as suitable for meta-analysis. Statistical heterogeneity of I^2^ = 25% was considered low, 50% moderate, and 75% high [36].

Sensitivity analyses were performed to assess the robustness of the outcomes by excluding the studies rated as ‘poor’ on the quality appraisal tool. Narrative synthesis was used to summarise and explain the conclusions across the studies.

## 3. Results

### 3.1. Study Selection

**Figure 1:**
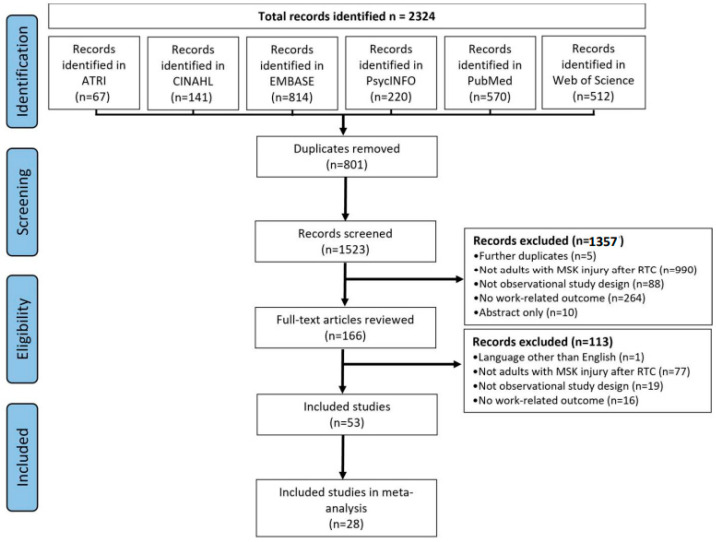
PRISMA flowchart.

Figure 1 shows that the electronic search identified 2324 studies. After removing 801 duplicates, 1523 records were screened on title and abstract with 166 included for full-text review. Finally, 53 studies were included in the narrative synthesis and 28 studies were included for meta-analysis. Five pairs of studies (*n* = 10) used (partially) the same participant cohorts [29,30,37,38,39,40,41,42,43,44]; all studies were kept in the narrative review. Of the ten studies, four were included in meta-analyses of data that were not repeated in their paired study [29,39,40,43], and in the case of Gopinath and colleagues [41,42], the 2015 study was used in the meta-analysis of RTW as more detailed data were reported in that study. When multiple articles from the same study reported the same work-related outcome measure, the article with the highest number of participants was used in the meta-analysis and the other articles were excluded from the meta-analysis to avoid overlap in participants [38,39,42,44].

### 3.2. Study Characteristics

The main characteristics of the included studies are described in Table 1. Of the 53 studies, 33 had a prospective cohort design, 13 were retrospective studies, and 7 studies had a cross-sectional design. The sample size varied from 11 [45] to 32,970 [30] participants. Most studies were conducted in Europe (*n* = 22, 42%), followed by Australia (*n* = 17, 30%), North America (*n* = 10, 19%), South America (*n* = 2 Brazil and Chile, 4%), Africa (*n* = 1 Uganda, 2%) and the Middle East (*n* = 1 Israel, 2%). Participants were sampled through various means including insurance companies and the general public (Table 1).

Of the *n* = 48 participant cohorts across the *n* = 53 included studies, most investigated patients with whiplash injuries (29/48, 60%), various musculoskeletal injuries (e.g., low back pain, neck pain, lower limb and hip fractures) (16/48, 34%), and mixed cohorts consisting of whiplash and other musculoskeletal injuries (3/48, 6%). The follow-up assessments from time of injury ranged from 3 days [46] to 8.5 years [47].

**Table 1:** Study characteristics of included studies.

First Author and Year	Country	Study Design and Size	Study Setting	Age (Years)	Sex (Female *N* (%))	Injury Type	Assessment Time (Baseline, Follow Up)	Specific Outcome Measures
Ackland 2013 [48]	Australia	Prospective cohort, 178	Hospital based	Median 35 (IQR 25–48)	72 (44.0%)	Neck pain	Baseline, after discharge, 6 m, 12 m	RTW: return to full duties, categorical (*n*, %) Work capacity: period of modified work, categorical (*n*, %) Work ability: categorical (*n*, %)
Barbosa 2014 [49]	Brazil	Cross-sectional, 210	General public	Aged between 18 and 35 years *n* = 59; aged between 36 and 45 years *n* = 64; age >45 years *n* = 42	Men only	Facial trauma	One time	Sick leave: categorical (*n*, %) Productivity loss: absenteeism, categorical (*n*, %)
Berecki-Gisolf 2013 [7]	Australia	Retrospective cohort, 5970	Transport Accident Commission	24.0% aged between 35 and 44	2208 (37.0%)	MSK or orthopaedic	Once (17 months after accident)	Sick leave: compensated days off work, categorical (%)
Biering-Sorensen 2014 [50]	Denmark	Prospective cohort, 7780 (104 WAD; 3204 other MSK)	Municipalities	WAD mean 36; MSK mean 42	WAD 75.0%; MSK 52.0%	Whiplash and other MSK pains	At 26 weeks, 1 year, 2 years, 3 years	RTW: categorical (*n*, %) Sick leave: number of participants sick listed, categorical (*n*)
Borchgrevink 1996 [47]	Norway	Retrospective cohort, 426	ED	Men median 37 (IQR 28–47); women 36 (IQR 26–48)	Men 174 (41.0%); women 252 (59.0%)	Whiplash	Prior to injury, follow up after injury (between 2.5 and 8−5 years)	RTW: categorical (*n*) Sick leave: registration for sick leave, categorical (*n*, %)
Brison 2000 [51]	Canada	Prospective cohort, 353	EDs	Aged 18–30 year *n* = 140 (39.7%); aged 31–50 years *n* = 150 (42.5%); aged 51–70 years *n* = 63 (17.8%)	224 (63.5%)	Whiplash	Baseline, 1, 2, 3, 6, 9, 12, 18, 24 months	Sick leave: missing <1 week of work, categorical (%); days missed work, continuous (mean) Work capacity: number of participants that modified work activities, categorical (%)
Buitenhuis 2009 [52]	The Netherlands	Prospective cohort, 879	Insurance company	Mean 36 (SD 12)	539 (61.3%)	Whiplash	Baseline, 6, 12 months	RTW: number of participants with no work or paid employment, categorical (*n*,%); working hours per week, continuous (mean, SD) Sick leave: people on work disability, categorical (*n*, %).
Bunketorp 2002 [53]	Sweden	Retrospective cohort, 108	EDs in 2 main hospitals	Mean 52 (SD 13)	66 (61.0%)	Whiplash	Once (17 years after injury)	Sick leave: number of participant on sick leave, work disability, or early retirement, categorical (%) Work ability: assigned medical disability, categorical (*n*, %)
Bylund 1998 [54]	Sweden	Prospective cohort, 255	General public	Women mean 33; men mean 31	132 (52.0%)	Cervical strain/ fracture	During the 2-year period from Jan 1, 1990 to Dec 31, 1991	Sick leave: categorical (*n*, %) per collusion mechanism; total sick leave days.
Carroll 2012 [55]	Canada	Prospective cohort, 5163	Claimants general public	Mean 39 (SD 15)	3479 (66.9%)	Whiplash	Baseline, 6 weeks, 3, 6 months	Sick leave: participants off work, categorical (*n*, %)
Casey ^1^ 2011 [38]	Australia	Prospective cohort, 246	Private NSW insurers	Mean 43 (SD 15)	78.0%*—n not reported*	Whiplash	Once (within 3 months of accident)	Work capacity: number of people unable to continue preinjury work capacity, categorical (*n*, %)
Casey ^1^ 2015 [37]	Australia	Prospective cohort, 246	Private NSW insurers	Mean 43 (SD 15)	78.0%*—n not reported*	Whiplash	Once (within 3 months of accident)	Work capacity: categorical (*n*, %)
de Rome 2012 [56]	Australia	Prospective cohort, 212	Hospitals and motorcycle repair service	24.7% aged between 17 and 25; 44.5% aged between 26 and 39; 30.8% aged between 40 and 75	NR	Fractures and cuts and abrasions	Baseline, 2, 6 months	Sick leave: days of work, continuous (mean)
Dufton ^2^ 2012 [39]	Canada	Retrospective cohort, 5581	Rehabilitation clinics	Mean acute 36 (SD 11); early chronic 36 (SD 11); chronic 36 (SD 11)	Acute 55.9%; early chronic 54.9%; chronic 61.6%*-n not reported*	Whiplash	At presentation, at discharge	Sick leave: participants off work, categorical (%) Work capacity: participants that modified work duties, categorical (%)
Dufton ^2^ 2006 [40]	Canada	Retrospective cohort, 2185	Rehabilitation clinics	Mean 36 (SD 11);	1208 (55.3%)	Whiplash	At presentation, at discharge	RTW: participants that returned to work, categorical (*n*, %); days to RTW, continuous (mean)
Ettlin 1992 [46]	Switzerland	Prospective cohort, 21	Emergency department	Mean 29	18 (86.0%)	Whiplash	Baseline, 3 days, 3 months, 1, 2 years	RTW: return to part-time and full-time employment, categorical (*n*)
Geldman 2008 [57]	England	Prospective cohort, 102	Police physiotherapy and rehabilitation department	Mean 34 (SD 7), range 19–51	35 (34.0%)	Whiplash	Baseline, 3, 12 months	RTW: participants that returned to usual work, categorical (%)
Gopinath ^3^ 2015 [42]	Australia	Prospective cohort, 284	MMA Personal Injury Registry database	Mean 46.4 (SD 17.1)	183 (64.4%)	MSK injuries (whiplash/ fractures)	Baseline, 12, 24 months	RTW: categorical (*n*, %) Work capacity: resumed to full or modified duties, categorical (*n*, %)
Gopinath ^3^ 2017 [41]	Australia	Prospective cohort, 284	SIRA Personal Injury Registry database	NR	NR	MSK injuries (whiplash/ fractures)	Baseline, 12, 24 months	RTW: categorical (*n*, %) Work capacity: resumed to full or modified duties, categorical (*n*, %)
Gray ^4^ 2018 [29]	Australia	Retrospective cohort, 24311	Transport Accident Commission	22.0% aged 15–24; 25.1% aged 25–34; 22.7% aged 35–44; 19.3% aged 45–54; 11.0% aged 55–70.	36.9%	Various MSK injuries	NA	RTW: failed and sustained RTW, categorical (*n*, %)
Gray ^4^ 2018 [30]	Australia	Retrospective cohort, 32970	Transport Accident Commission	22.0% aged 15–24; 25.1% aged 25–34; 22.7% aged 35–44; 19.1% aged 45–54; 11.0% aged 55–70.	36.0%	Various MSK injuries	NA	Work capacity: Gradual RTW pathway, categorical (*n*., %) and days (median, IQR)
Guest 2017 [58]	Australia	Retrospective cohort, 6341 (MSK *n* = 5734; MSK + psychological distress (MSKPD) *n* = 607)	SIRA Personal Injury Register	MSK group mean 43 (SD 16); MSKPD group mean 44 (SD 16)	MSK group 3317 (58.0%); MSKPD group 395 (65.0%)	Various MSK injuries	Once	Productivity loss: economic loss claim, categorical (*n*,%)
Gun 2005 [59]	Australia	Prospective cohort, 135	EDs, medical and physio practices	Mean 36 (SD 15)	98 (83.0%)	Whiplash	Baseline, 6 weeks, 12 months	RTW: categorical (*n*, %)
Herrström 2000 [60]	Sweden	Prospective cohort, 158	Health care-based register	Range 10–77; women median 30; men median 35	74/125 (59.0%) (reported for 12 month data *n* = 125)	Whiplash	Baseline, 12 months	Sick leave: reported sick leave, categorical (*n*, %); duration sick leave (mean, range)
Hildingsson 1990 [61]	Sweden	Prospective cohort, 93	University based	Median 31 (range 17–67)	53 (57.0%)	Whiplash	Baseline, follow up (mean 25 months)	Sick leave: reported sick leave, categorical (*n*) Work capacity: changes in work status, categorical (*n*)
Holm 1999 [62]	Sweden	Cross-sectional, 4121	Road Traffic Injury Commission	WAD 1989: partial or full work disability (*n* = 172) mean 47 (SD 10); no work disability (*n* = 106) mean 39 (SD 11) WAD 1994: partial or full work disability (*n* = 417) mean 43 (SD 10); no work disability (*n* = 277) mean 37 (SD 11)	WAD 1989: partial or full work disability 100/172 (57.6%); no work disability 71/106 (67.0%) WAD 1994: partial or full work disability 256/417 (61.4%); no work disability 156/277 (56.3%)	Whiplash	Once	RTW: return to full work capacity, categorical (%) Work capacity: partial and full work disability, categorical (*n*, %)
Hours 2014 [31]	France	Prospective cohort, 253	Registry of Road Crash Trauma	Age group 16–24: grade I: *n* = 19 (30.6%), grade II *n* = 35 (32.1%); age group 25–34: grade I *n* = 14 (22.6%), grade II *n* = 32 (29.4%); age group 35–44: grade I *n* = 15 (24.2%), grade II *n* = 25 (22.9%); age group 45–54: grade I *n* = 2 (3.2%), grade II *n* = 9 (8.3%); age group 55 + : grade I *n* = 12 (19.4%), grade II *n* = 8 (7.3%)	157 (62.0%)	Whiplash	Baseline, 12 months	RTW: participants that did not RTW, categorical (%) Sick leave: categorical (*n*, %) and (median, IQR).
Hoving 2003 [63]	Australia	Cross-sectional, 71	Physio, GP, rheumatology clinics	Mean 40 (SD 14)	59 (83.1%)	Whiplash	Once	Work ability: Neck Disability Index—Work Item mean and The Northwick Park Neck Pain Questionnaire-Work/housework, continuous (mean, SD); problem elicitation technique (PET)—work for wages, categorical (*n*, %)
Kasch 2001 [64]	Denmark	Prospective cohort, 141	General public	Mean 36 (SD 11);	74 (52.0%)	Whiplash	Baseline, 1 week, 1, 3, 6, 12 months	RTW: participants that did not RTW, categorical (*n*, %) Work capacity: returned to modified job functions, categorical (%)
Kasch 2011 [65]	Denmark	Prospective cohort, 141	EDs	NR	NR	Whiplash	At 1 week, 1, 3, 6, 12 months	RTW: categorical (*n*, %)
Kasch 2019 [66]	Denmark	Prospective cohort, 143	EDs	Mean 35.8 (SD 10.5)	75 (52.0%)	Whiplash	At 1 week, 6, 12 months	Work capacity: participants ‘recovered’ (*n*, %)
Kinzel 2006 [45]	Australia	Prospective cohort, 11	Hospital based	Mean 34 (range 16–67)	1 (9.0%)	Upper limb MSK injuries	Baseline, regular outpatient appointments up to 12 months	RTW: categorical (*n*)
Krogh 2018 [67]	Denmark	Prospective cohort, 141	EDs	Mean 36 (SD 11)	74 (52.0%)	Whiplash	Baseline, 1, 3, 6, 12 months	Work capacity: participants’ recovery status, categorical (*n*, %)
Leth-Petersen 2009 [68]	Denmark	Retrospective cohort, 1203	Records of National Board of Industrial Injuries	Women-comp mean 36 (SD 9); women-noncomp 36 (SD 9); men-comp 35 (SD 8); men-noncomp 36 (SD 9)	844 (70.0%)	Whiplash	Retrospective data	Productivity loss: percentage of lost earnings capacity, categorical (*n*, %)
Mankovsky-Arnold 2017 [69]	Canada	Cross-sectional, 105	Rehabilitation clinics	Overall mean 37 (range 17–57); men mean 38 (SD 9); women 36 (SD 11).	49 (47.0%)	Whiplash	Once	RTW: employment status, categorical (*n*, %)
Miettinen ^5^ 2004 [43]	Finland	Prospective cohort, 182	Insurance companies	Overall mean 42; men 45 (SD 15); women 40 (SD 14)	117 (64.3%)	Whiplash	Baseline, 12 months	Sick leave: (length of) sick leave, categorical (*n*, %)
Miettinen ^5^ 2002 [44]	Finland	Prospective cohort, 201	Insurance companies	Mean 42	NR	Whiplash	Baseline, 12 months	Sick leave: (length of) sick leave, categorical (*n*, %)
Munjin 2011 [70]	Chile	Retrospective cohort, 46	Hospital based	Mean 49 (range 16–70)	37 (80.4%)	Spine fracture (no neurologic impairment)	Retrospective data	Sick leave: leave of absence, continuous (average, median, range)
Myrtveit 2015 [71]	Denmark	Prospective cohort, 740	Clinics in University Hospitals	Mean 35 (SD 11)	475 (64.1%)	Whiplash	Baseline, 12 months	Work ability: participants that reported reduced work capability, categorical (*n*, %)
Nguyen 2019 [32]	Australia	Prospective cohort, 498	EDs, rural health services, primary care and the NSW State Insurance Regulatory Authority—Personal Injury Registry, Claims Advisory Service.	Neck injuries: mean 40.2 (SD 16) Lower back injuries: mean 35.7 (SD 17) Lower limb injuries: mean 38.9 (SD 15)	Neck injuries: 107 (64.5%) Lower back injuries: 31 (39.7%) Lower limb injuries: 78 (30.7%)	Various MSK injuries	Baseline, 6 months	RTW: categorical (*n*, %)
O’Hara 2018 [72]	Uganda	Prospective cohort, 57	Hospital based	Median 34 (IQR 27–45)	9 (11.1%)	Isolated tibial/femoral fracture	Baseline (within 48 hrs of hospital admission), 6, 12, 24 months	RTW: categorical (*n*, %); time to RTW, continuous (mean, 95%CI) Productivity loss: reduction in monthly income, continuous (mean difference, 95%CI); debts, continuous (mean, 95%CI)
Pieske 2010 [73]	Germany	Prospective cohort, 81	Hospital based	Mean 33.0 (SD 12.0), range 18–74	45 (55.6%)	Whiplash	Baseline, 1, 3, 6 months	RTW: inability to work, categorical (*n*, %) Sick leave: duration of not being at work, categorical (*n*, %)
Prang 2015 [74]	Australia	Cross-sectional, 1649	General public	Mean 44 (SD 15).	685 (41.5%)	Various MSK injuries	Once	RTW: categorical (*n*, %)
Ratzon 2015 [75]	Israel	Cross-sectional, 123 (Whiplash n = 76; Hip *n* = 47)	Outpatient clinics and hospital	Whiplash mean 33 (SD 11.8); hip 38 (12)	Whiplash 40 (52.0%); hip 12 (25.5%)	Whiplash and hip injury	Once	Work ability: degree of disability (based on medical files), categorical (*n*, %)
Rebbeck 2006 [76]	Australia	Prospective cohort, 114	Insurance databases	Mean 39.4 (SE 1.3)	Gender female *n* = 79 (69.3%), male *n* = 35 (30.7%).	Whiplash	Baseline (at 3 months), 6 months, 2 years	Sick leave: participants taking days off work, categorical (*n*, %); number of days taken off work, continuous (median, IQR)
Rosenthal 1979 [77]	USA	Retrospective cohort, 43	University hospital	NR	NR	Hip fracture/dislocation	Retrospective data	RTW: not returning to work, categorical (*n*, %)
Sarrami 2016 [78]	Australia	Retrospective cohort, 90	SIRA Personal Injury Register	Mean 46 (SD 12), range 23–73	48 (53.0%)	Spine surgery	Retrospective data	RTW: categorical (%) Work capacity: return to pre-injury duties, categorical (%)
Schreiber 2009 [79]	USA	Prospective cohort, 38	Private chiropractic offices	Mean 37	“male to female ratio nearly 1:1”	Whiplash	Once	RTW: employment, categorical (%) Work ability: decreased work function, categorical (*n*)
Scuderi 2005 [80]	USA	Prospective cohort, 270 (Workers Compensation (WC) group *n* = 54; Personal Injury (PI) group *n* = 216)	Workmen’s Compensation System	WC group mean 43 (range 25–62); PI group mean 35 (range 18–68)	WC group 20 (37.0%); PI group 112 (51.9%)	Neck pain	Baseline (referral to surgeon), follow ups until considered RTW/ reaching max improvement/ lost to follow up after 2 years	RTW: unable to RTW, categorical (*n*) Sick leave: lost days of work, continuous (total, mean)
Smed 1997 [81]	Denmark	Prospective cohort, 29	University hospital	Median 33 (range 22–56)	17 (58.6%)	Whiplash	1 month post-injury	Sick leave: categorical (*n*)
Swartzman 1996 [82]	Canada	Retrospective cohort, 62 (litigants *n* = 41, post-litigants *n* = 21)	Private practice, University hospital	Litigants mean 38; post-litigants mean 39	Litigants 31 (76.0%); post-litigant 17 (81.0%)	Whiplash	Once	RTW: employment status, categorical; number of hours per week employed outside the home, continuous (mean)
Virani 2001 [83]	Canada	Cross-sectional, 356 (physicians *n* = 149; nonphysicians *n* = 207)	University hospital	Physicians mean 46; nonphysicians mean 40	NR	Whiplash	Once	Sick leave: time off, categorical (%)
Vos 2008 [84]	The Netherlands	Prospective cohort, 42	GPs	Mean 35	NR	Neck pain and whiplash	Baseline, 6, 12, 26, 52	Sick leave: categorical (%)

Superscript numbers (^1,2,3,4,5^) attached to author names indicate the pairs of studies that report on the same cohort of participants. Abbreviations: IQR, interquartile range; MSK, musculoskeletal complaints; NR, not reported; RTW, return to work; SE, standard error; WAD, whiplash-associated disorder.

Four studies were included in which adolescents were included in the study cohort [29,30,31,32]. Two studies shared the same cohort, 22% of which was aged 15–24 years [29,30]. One study presented a cohort in which 32% of people with whiplash were aged 16–24 years [31]. One study included participants aged at least 17 years but the mean (SD) age was between 35.7 (17) and 40.2 (16) years depending on injury location [32]. Specific details concerning the exact number of participants who were aged <18 years could not be ascertained from the authors. Overall, adolescents only consisted of a small portion of the total study populations, and as such these studies were not excluded from the review [32].

### 3.3. Quality Appraisal

Inter-rater agreement for quality appraisal of individual studies was substantial (κ = 0.70, *p* < 0.001) with 602/742 agreements [34].

Results of the quality appraisal of the individual studies are presented in Table 2. Twenty-one studies were of good quality (40%), 16 studies of fair quality (30%) and 16 studies of poor quality (30%). The majority of studies clearly defined the research question (*n* = 45, 85%), clearly specified and defined the study population (*n* = 48, 91%), selected or recruited participants from same or similar populations during the same time period (*n* = 49, 92%), and had a timeframe that was sufficient to reasonably expect to see an association between exposure and outcome (*n* = 43, 81%) (Table 2). However, 44 studies did not provide a sample size justification, power description or variance and effect estimate (83%), and 17 studies did not measure or adjust for confounding variables for their impact on the relationship between exposure and outcomes (32%). Thirty-five studies did not measure the exposure variable more than once over time (*n* = 35/40, 88%).

### 3.4. Work-Related Outcomes

Out of all included papers (*n* = 53), outcomes related to RTW were reported by 27 studies (51%;), sick leave by 23 studies (43%), work capacity by 13 studies (25%), work ability by 6 studies (11%) and productivity loss by 4 studies (8%) (Supplementary Material S2). Pooled prevalence and quality of evidence are summarised according to outcomes of RTW, sick leave and work capacity. Meta-analysis was not possible for work ability and productivity loss.

#### 3.4.1. Return to Work

**Figure 2:**
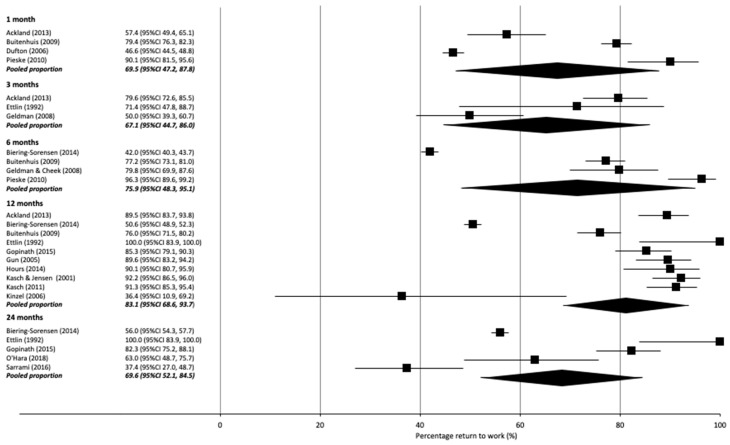
Forest plot with percentages and pooled percentage of RTW as reported per month.

RTW proportions were reported at different time points, ranging from 1 week to 3 years (Table 3). Six studies did not specify a specific RTW time point (Table 3) [29,47,62,74,77,80]. Pooled RTW percentages showed a RTW prevalence of 69% (95% CI 47, 88, I^2^ 99%; 4 studies) up to 1 month, 67% (95% CI 45, 86, I^2^ 91%; 3 studies) up to 3 months, 76% (95% CI 48, 95, I^2^ 99%; 4 studies) up to 6 months, 83% (95% CI 69, 94, I^2^ 99%; 10 studies) at 12 months and 70% (95% CI 52, 85, I^2^ 96%; 5 studies) at 24 months (Supplementary Material S3; Figure 2). Pooled percentages at all time points had high statistical heterogeneity (>91%) (Supplementary Material S3).

#### 3.4.2. Sick Leave

Sick leave was reported as percentages for a certain duration and as percentages reported at a certain time point (Table 4). Three studies did not report a specific time point or duration (Table 4) [7,39,83].

**Figure 3:**
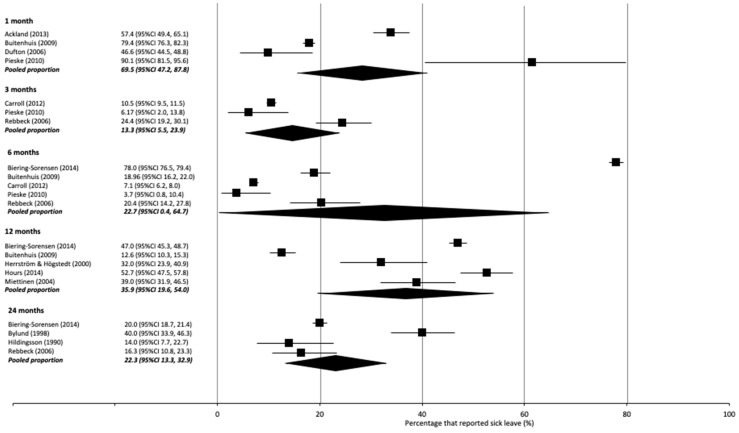
Forest plot with percentages and pooled percentage of participants taking sick leave as reported per time point (1, 3, 6, 12 and 24 months).

Sick leave was reported at different time points, ranging from 1 month to 2 years (Table 4). Pooled percentages revealed that 27% (95% CI 16, 41; I^2^ 97%; 4 studies) of injured workers had used sick leave at 1 month post-RTC, 13% (95% CI 6, 24; I^2^ 94%; 3 studies) at 3 months, 23% (95% CI 0, 65; I^2^ 100%; 5 studies) at 6 months, 26% (95% CI 20, 54; I^2^ 99%; 5 studies) at 12 months and 22% (95% CI 13, 33; I^2^ 94%; 4 studies) at 24 months (Supplementary Material S4; Figure 3).

**Figure 4:**
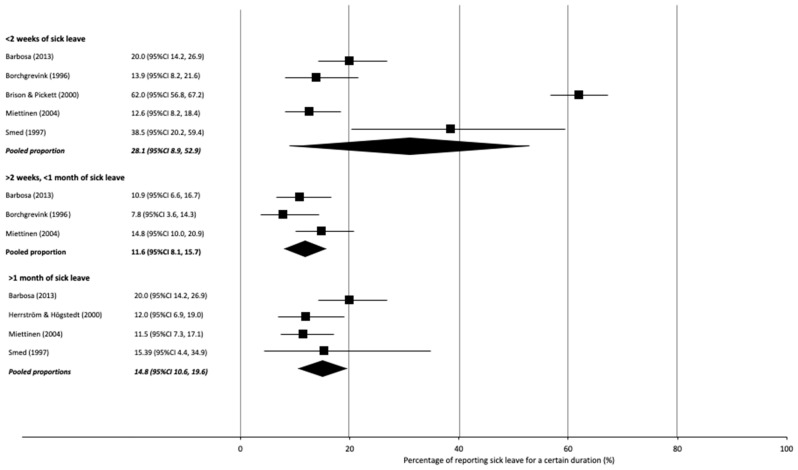
Forest plot with percentages and pooled percentage of participants reporting sick leave for a certain duration.

Pooled sick leave of less than 2 weeks was reportedly used by 28% of injured workers (95% CI 9 to 53; I^2^ 98%; 5 studies), between 2 and 4 weeks by 12% (95% CI 8, 16; I^2^ 41%; 3 studies), and sick leave of more than 4 weeks was reported by 15% (95% CI 11, 20; I^2^ 46%; 4 studies) (Supplementary Material S4; Figure 4). Injured workers in two studies reported a mean sick leave duration of 6.6 days (no estimate of variance reported) [51] and 13.5 days (SD 29.8) [56] at 6 months post-RTC. Another study reported a duration of 2–3 weeks of sick leave at 12 months [60], and a retrospective study reported a mean leave of absence of 104 days (range 24–382) [70]. Further information regarding how sick leave was measured and reported is available in Supplementary Material S2.

#### 3.4.3. Work Capacity

Eleven studies (thirteen publications) reported outcomes related to modifying duties at work and/or the inability to work [61] for time periods up to 6 months post-RTC [51] (Supplementary Material S2). One study reported that 38% of participants were unable to continue pre-injury work within 3 months of injury [38] and the pooled percentage from two other studies showed that 10% of participants did not return to pre-injury work capacity at 1 year post-RTC (pooled percentage 10%, 95% CI 1 to 20) [66,67] (Supplementary Material S2). Gopinath et al. (2015) reported that 82% of participants resumed full duties at 12 months and 89% at 24 months [42]. In contrast, in a study with a lower RTW rate (37%) at 2 years, 23% had returned to pre-injury duties at 2 years [78]. Other studies report periods of modified duties [48], rates of modified duty use [51], and results by injury chronicity [39] (see Supplementary Material S2).

#### 3.4.4. Work Ability

Six studies [48,53,63,71,75,79] described outcome measures consistent with work ability. There was no consistency of measurement tools between the six studies, and they were rarely validated, as such a meta-analysis was not conducted. Instead findings are reported narratively.

Percentage of participants with reduced work ability, defined as ‘capability’ (based on a combination of sick leave and reduced work hours) was 15% at 12 months in one study [71]. When reduced work ability was defined as ‘decreased work function’ (self-reported limitation in ability to work) [79], the prevalence was 34% at mean 4.5 months (*n* = 38) (Supplementary Material S2). Bunketorp and Carlsson used insurance physicians to assign a degree of medical disability resulting from crash-related neck injury to 18 of 25 participants who had residual neck pain and an insurance claim [53]. When functional capacity evaluations were combined with medical chart review in the definition of work ability, 26 of 76 patients (34%) with WAD were rated as having work disability at mean 2.4 (SD 22) years (range 0.4–13 years) [75].

Hoving and colleagues (2003) [63] reported results in a cross-sectional study for the work-specific items within the Neck Disability Index (mean (SD) 2.2 (1.3) where 0 = no disability and 5 = total disability) and Northwick Park Neck Pain Questionnaire (mean (SD) 1.7 (1.2) where 0 = no difficulty and 4 = severe difficulty) in people with WAD from 0 to >24 months post-injury. Patients with neck pain were asked to nominate their reasons for their delay in returning to full work duties in a study by Ackland and colleagues [48]. Of the 69% of patients who did experience a delay, 50% nominated their neck injury or neck pain as the reasons, whilst 12% nominated other crash-related injuries, and 7% nominated psychological issues.

#### 3.4.5. Productivity Loss

Four studies reported measures of health-related productivity loss [49,58,68,72]. Absenteeism was reported by one study in *n* = 13/15 participants with facial injuries [49]. Those who experienced psychological distress in conjunction with their musculoskeletal injury had statistically similar rates (*p* = 0.96) of not filing for an economic loss claim (*n* = 329, 54.2%) compared with those who had musculoskeletal injury only (*n* = 3101, 54.1%) [58]. At 2 years post-RTC, monthly income was 62% less than pre-injury monthly earnings (mean difference, USD$117.50; 95%CI USD$34 to USD$201) for those with tibial/femoral fractures in Uganda [72]. Of 1203 participants with whiplash in Denmark, 47% experienced lost earnings capacity in the 5 years following RTC [68] (see Supplementary Material S2).

### 3.5. Sensitivity Analysis of Work-Related Outcomes

Return to work outcomes were robust to sensitivity analysis (i.e., excluding studies rated as ‘poor’) for 1, 3, 6, and 24 months but not for 12 months (all studies 83.05%, moderate and good quality studies 80.62%, *p* < 0.01) (Supplementary Material S5). Sick leave duration was robust to sensitivity analysis, except for sick leave duration < 2 weeks (all studies 28.10%, moderate and good quality studies 13.37%, *p* < 0.001) (Supplementary Material S6). Sick leave percentages reported at 6 and 12 months were robust to sensitivity analysis, but not 1 month (27.32% vs. 20.40%, *p* < 0.001) and 3 months (13.32% vs. 9.76%, *p* < 0.001) (Supplementary Material S6). Three out of four studies at 24 months were rated as poor quality, therefore sensitivity analysis was not possible.

### 3.6. Factors Associated with Work-Related Outcomes

Fifty-six percent of all studies (*n* = 30/53) reported information on (potential) factors associated with work-related outcomes (Supplementary Material S7). Of the studies that reported RTW, 52% (*n* = 14/27) investigated associated factors. For sick leave, 39% (*n* = 9/23) investigated associated factors, for work capacity 23% (*n* = 3/13), for work ability 50% (*n* = 3/6), and for productivity loss 75% (*n* = 3/4) of studies investigated associated factors (Supplementary Material S7).

#### 3.6.1. Return to Work

Risk factors for delayed return to work were predominantly health-related including injury type (whiplash, dislocations vs. limb fracture), greater severity of injury, being admitted to hospital, having a higher baseline OMPSQ score, having lower baseline mental health-related quality of life, lower pre-injury fitness levels and the presence of a pre-injury chronic illness [29,32,42,57]. Sociodemographic characteristics such as lower socioeconomic status, lower education and being a widow, separated or divorced were also risk factors for delayed return to work [30,74]; however, the findings for age and sex on return to work were mixed [30,42,74]. Support from employers was positively associated with returning to work [74]. Consulting a lawyer was associated with a 5-fold lesser chance of returning to work at 1 year, but this association was not significant after adjusting for the neck pain outcome score, bodily pain scores and role emotional scores [59].

#### 3.6.2. Sick Leave

Two studies reported associations between taking time off work/sick leave and being female [7,54] and two studies reported that women took longer average sick leave than men [44,54]. Longer sick leave was reported in participants who were married, divorced or widowed compared to single persons (*p* < 0.05) [43]. No significant associations on the length of sick leave were found in Miettinen et al. [43] with age, education, speed of the vehicle, position in the car or use of the seatbelt. Receiving workers compensation for neck pain after RTC was associated with more days off work compared with seeking compensation via a personal injury claim [80].

Four studies investigated the relationship between injury-related factors and sick leave. Sustaining a whiplash injury was a risk factor for taking more sick leave [54] and not recovering from a whiplash injury was a risk factor for being on sick leave [53], although one study found no difference in length of sick leave between those without whiplash, with grade 1 or with grade 2 whiplash [31]. Level of protective equipment worn by motorcycle riders (unprotected, partial, or full protection) was not related to number of days off work [56].

#### 3.6.3. Work Capacity

One study reported that those who were unable to continue in their pre-injury work capacity scored significantly worse on all health outcome measures including the SF-36 and pain catastrophizing scale [37], whereas another study reported that there were no significant associations between work capacity and potential predictors including age, gender, surgery type and location, and their socioeconomic indexes area [78]. Holm et al. [62] reported that all participants with partial or full work disability were over 40 years of age, had over 15% of medical impairment and a lower professional status than those with no work disability.

#### 3.6.4. Work Ability

One study reported no evidence of an association between gender and status as work disabled (*p* = 0.12) [53]). As reported by Myrtveit et al. [71], reduced work capacity was associated with preferring to take medications, sickness absence, being referred to a physiotherapist or chiropractor; whereas participants who believed the active coping preferences of “living as usual” and “change of lifestyle” could make them better were protective for reduced work capacity.

#### 3.6.5. Productivity Loss

Barbosa et al. [49] reported a significant association between the occurrence of a facial injury and absenteeism (*p* = 0.024). An economic analysis of data from Denmark found those with WAD injuries who were awarded compensation on the basis of injury severity were more likely to have lost earning capacity in the long term [68]. O’Hara et al. [72] reported no association between surgical treatments, monthly income, debt, employment or dependents (Supplementary material S7).

### 3.7. Impact of Deviation from Protocol on Results

As described in the methods Section 2.2.1, four studies that included adolescents in their cohorts were included in this review as a deviation from the study protocol [29,30,31,32]. The four studies that were included with adolescent participants did not limit the collection of RTW data within their study to adults (>18 y) only, and results were not presented separately for adolescents and adults, meaning data could not be extracted for adults only. If the adolescents were not previously working prior to their RTC, they were not included as having RTW in these studies; and if they were employed prior to their RTC, their post-injury RTW status is also of interest. These studies treated any adolescents in their cohorts as adults; this is evident in the manner in which the cohort characteristics were described. The presentation of the cohort characteristics does not clearly state what percentage of the cohort was represented by adolescents; however, it is likely to be small. This does restrict us from making any comparison across key confounders or outcomes between adolescents and the adults in the wider review. Two studies by Gray and colleagues [29,30], based on the same dataset, reported 22% of their cohort was aged 15–24 y, and mean age is not given. The study by Hours and colleagues [31] reported 21% of their cohort was aged 16–24 y, and mean age is not given. The study by Nguyen and colleagues [32] included *n* = 498 total from the age of 17 y and above, with a mean (SD) age of 40.2 (16) y for neck injuries, 35.7 (17) y for lower back injuries, and 38.9 (15) for lower limb injuries. Any 17 y old participants would have been more than 1 standard deviation outside of the mean. In a normally distributed dataset, 68% of the cohort is within one standard deviation of the mean (mean +/−1 SD).

The potential impact of including these four studies on the pooled meta-analyses is minimal. Only one study [32] contributed data to meta-analyses (see Figure 2 and Figure 3). Results from this study by Nguyen and colleagues [32] for the rate of RTW at 12 months was consistent with several other studies in the meta-analysis [59,65,66]. The use of sick leave at 12 months was highest in this study across the five studies in this meta-analysis, however likely balanced out in the meta-analysis by a corresponding low usage rate from another study [52].

## 4. Discussion

This systematic review identified 53 observational studies in which RTW, sick leave, work capacity, work ability, and health-related work productivity loss were measured in adults with a musculoskeletal injury after RTCs. Findings from the meta-analyses within this review should be interpreted with caution, as the degree of statistical heterogeneity was high. Each pooled statistic was the product of the combination of different studies, and represents a combined estimate based on similar data drawn from at least two studies. Outputs from those meta-analyses demonstrated the pooled RTW rate generally increases post-injury from 70% of injured persons having RTW at 1 month, to 67% at 3 months, 76% at 6 months, and 83% at 12 months. These findings suggest that most injured persons are back at work within a month of their RTC, and of those who are not, some go on to return gradually to work over the first year after RTC, whilst some will not have a successful RTW at 1 year post-injury. Approximately one-third of injured workers used sick leave in the year after RTC, and approximately one-sixth used more than 4 weeks of sick leave. Using sick leave may be the best (or only) option for individuals without suitable modified duties available to them in their workplace. Results of four studies demonstrated a varying percentage (though always <50%) of workers used modified duties at work. Few studies identified by this review measured work ability (6 of 50 studies, 12%) or health-related work productivity loss (4 of 50 studies, 8%). More research is needed in this client population regarding these outcome measures for appropriate conclusions to be drawn.

There are several key aspects of the methodology of the included studies that are worth highlighting, and should be considered with the findings. It is encouraging to have a high percentage of prospective observational studies within this review, and many with large sample sizes from administrative databases, which can both be considered as markers of methodological quality within the field. On the other hand, administrative databases may be biased representations of the total population of adults with musculoskeletal injuries after RTC, over-emphasising the experience of those who presented to hospital (ED/hospital datasets) or those who were eligible for compensation (insurance databases), depending on the jurisdiction in which they live. Regarding type of injury, the most common injury type were whiplash injuries. This fits with the frequency of whiplash injuries in RTC and the emphasis placed on these injuries in the literature [1,85,86,87]. It must be acknowledged that as a result the work-related findings of this review are most relevant to those with whiplash injuries and are less generalizable to musculoskeletal injuries as a whole. Developed countries such as Canada, Australia and Denmark may also be overrepresented in the results—only three studies were from the developing nations [88] of Uganda [72], Brazil [49], and Chile [70]. A report published by the Global Road Safety Facility in 2014 described an annual total of 78.2 million non-fatal injuries in less developed regions such as Sub-Saharan Africa [89], therefore future research on the employment impacts of RTC are needed from these regions to better represent the global burden of traffic injuries. Many studies did not justify their sample size, either with a power calculation or an indication of what percentage of eligible individuals consented to participate. Absence of *a priori* sample size calculations in observational studies has implications for detecting a difference in the primary outcome between sub-groups. In addition, many studies did not provide an indication of how representative their sample was of the true population of those injured by RTCs by omitting a comparison between those who consented versus those who declined to participate. However, the use of data such as the age and sex of those who declined to participate may not have been available to researchers, as there are ethical concerns related to the collection of data from those who have not consented.

Of the studies reporting RTW rates, the most data were available for 12 months post-injury, demonstrating that 83% of adults with a musculoskeletal injury after RTC had returned to work by 12 months. RTW rates range from 1 month (69.5%) to 12 months (83%) and suggests that those who have not returned by 1 month may take several months to achieve a successful RTW. Vocational rehabilitation programs can facilitate RTW by optimising work participation usually with a combination of medical, psychological, social and/or occupational strategies: for example, assessments of functional capacity and vocational goals, vocational counselling, training and work experience opportunities (particularly within the workplace), and assistance with job seeking [90]. Findings from this review suggest that there is a need for vocational rehabilitation services across the first year after RTC. Existing evidence from the field of occupational injuries cannot be assumed to apply to those injured in RTCs, as the legislated requirements for support from an employer after a work-related injury may differ to those following RTC-related injury.

Existing evidence for vocational rehabilitation, and rehabilitation more broadly, also needs to expand to encompass developing nations. The World Health Organization’s ‘Decade of Action for Road Safety 2021–2030′ calls for the provision of rehabilitation to all RTC injured persons, and particularly for “protections for people with disabilities to keep their jobs or be hired in new jobs through the provision of incentives for employers will further alleviate the socioeconomic consequences of permanent disability”[91]. Liability insurance could contribute greatly to this vision; for example in Uganda in 2020, a third party insurance compensation scheme was established to help victims of RTCs pay for medical expenses [92]. Road traffic injuries place a significant burden on the health care systems of developing nations, where additional socioeconomic consequences flow on from loss of employment and permanent disability [93].

Interestingly, the pooled data (5 studies) for 24 months post-injury resulted in the same RTW rate as one month post-injury—70%. This could reflect a portion of injured persons being unable to sustain their RTW, which is known to occur [29]. This could also be due to methodological issues with drop out such that individuals who have not yet RTW at 2 years may be more likely to have time to engage in research participation than those who have RTW. The needs of these individuals attempting to remain at work during their recovery are not well understood, and are worthy of further exploration.

Inconsistency in the definition of RTW, with no universally accepted standard, makes it difficult to carry conclusions across different types and mechanisms of injuries. In this review, some studies considered return to exactly the same job, hours and/or conditions (e.g., [48,57,62]), while other studies defined RTW as return to any form of paid employment, including different roles, reduced hours and modified duties (e.g., [42,46]). More detail and consistency in the definition of RTW are needed in future research studies. This definition may also reflect the method of data collection. Studies may use self-reported data regarding RTW, and therefore RTW becomes open to interpretation by the participant themselves if clear instructions are not provided by the investigators. While administrative databases are likely to provide more consistency in how RTW is defined, those definitions are still likely to differ between countries (e.g., Australia [29] vs. Denmark [68]).

Meta-analyses of sick leave data demonstrated approximately 1 in 4 injured persons used sick leave in the first year after their RTC, and for those who did use sick leave, 15% used it for more than 4 weeks. Musculoskeletal disorders in general are responsible for a significant amount of sickness absence and work disability, with the associated loss in productivity being equivalent to an estimated 2% of the European Union’s gross domestic product [94]. In a Swedish study of individuals who suffered severe physical trauma, 11% of individuals were on full-time sick leave 12 months after their injury [95]. In this review, the RTW rate at 1 month post-injury was 70%, and the proportion of injured persons using >4 weeks of sick leave was 15%. This may indicate a gap for ~15% of injured persons who are unable to work, and who may or may not be able to source other forms of income, such as government supportive payments or insurance payouts. Financial strain after RTC is a significant concern: some may find ongoing medical costs a cause of bankruptcy [96]. To avoid bankruptcy, some workers make the decision to RTW before their illness or injury has recovered [97]. Casualization of workforces [98] may be another potential reason behind the early RTW of those who are still ill/injured, and the ~15% of persons unable to RTW or to access paid sick leave. The jurisdiction in which an injured person resides is an important factor in determining access to financial support. For example, in Australia, the state of Victoria has a no-fault based scheme that pays benefits to injured persons in the form of wage replacement when they are unable to work [99]. In contrast, the state of Queensland has a fault-based scheme that does not pay wage replacements during the life of a claim, rather including income lost in the final claim amount [100]. In the present review, one study identified individuals with neck pain after RTW took more days off if they were receiving workers compensation in comparison to compensation via a personal injury claim [80]. More research into the relationship between fault status, compensation status, and work outcomes is needed.

A wide range of domains were assessed and found to be associated with the work-related outcomes within this review. Categories of independent variables included sociodemographic factors, psychosocial health, injury and RTC characteristics, pre-crash mental and physical health, educational attainment, employer support, and type of work. The significance of any association, and if present, the direction of the association varied for many of these characteristics across multiple work-related outcomes. There are some links that could be drawn from the results of the present review; for example, people who were married, divorced or widowed were more likely to have longer sick leave (vs single people) [43] and a delayed RTW (vs never married) [74]. Gender (or sex) was highly variable (and sometimes used interchangeably), being associated with taking sick leave [7,43,54], having no evidence for an association with work ability or work capacity [53,78], and having mixed results for an association with RTW [29,42]. The systematic review by Samborec and colleagues [101] examined the relationship between biopsychosocial factors and non-recovery following minor RTC-related injury. In this review, RTW was one of the many outcomes that could be considered as a measure of recovery. Similarly to the present review, there was conflicting evidence for the association of sex (female/male) with non-recovery [101]. The strongest evidence for an association with non-recovery was found for characteristics of pain (initial intensity, duration, severity) and pre-injury mental and physical health [101]. Results of both of these reviews highlight the multifactorial nature of recovery and RTW—recently supported by the outcomes of our Delphi study [102], and the difficulty in predicting when an individual will RTW. Support to RTW or to recover more broadly after RTC should therefore be individualised.

This review has a number of strengths. A thorough database search was conducted across five scholarly databases and one grey literature source specific to the transport industry. Independent review by two co-authors for screening, data extraction and appraisal led to high ratings of agreement. Results were presented by timeframe, to aid in the interpretation of recovery after RTC over time. Finally, five work-related outcomes were investigated, expanding the knowledge base beyond a focus on achievement of RTW.

There are also limitations that must be acknowledged. There were high levels of heterogeneity in the results of the meta-analyses. The database search was restricted to studies published in English which may have contributed to the under-representation of results from developing countries. There are a number of validated tools for measuring work capacity and work ability that this review was expecting to find [26]. However, few studies measured either of these concepts comprehensively and when they did, it was often to report a rate of work incapacity or inability defined to suit the individual study and lacking evidence of the psychometric properties of the outcome. Many of the studies included in this review recruited only patients with whiplash injuries post-RTC, making these results more applicable to injured persons with this particular musculoskeletal condition. On the whole, the cohorts included in this review could be said to represent mild to moderate musculoskeletal injuries, and not musculoskeletal injuries in general.

## 5. Conclusions

This review found that the pooled RTW rate increases in the first-year post-injury, from 70% at 1 month to 83% at 12 months. Approximately one-third of injured workers use sick leave in the year after RTC, and approximately one-sixth used more than 4 weeks of sick leave, reflecting a significant interruption to their employment and cost to their employer. Less than half of injured persons used modified duties at work following RTC-related musculoskeletal injury. More research is needed to understand the impact of RTC on work ability and health-related productivity loss. Clinicians are encouraged to consider the multiple potential factors of influence (health, sociodemographic, work-related) for a client’s recovery and RTW after RTC, and be familiar with the legal and insurance frameworks operating in their jurisdictions.

## Figures and Tables

**Table 2:** Quality assessment with the National Heart, Lung, and Blood Institute Study Quality Assessment Tools For observational cohort and cross-sectional studies.

Study	1. Was the Research Question Clearly Stated?	2. Was the Study Population Clearly Specified and Defined?	3. Was the Participation Rate of Eligible Persons At Least 50%?	4. Were All the Subjects from Similar Populations? Were Eligibility Criteria Prespecified and Applied Uniformly?	5. Was A Sample Size Justification, Power Description, or Variance and Effect Estimates Provided?	6. Were the Exposure(S) of Interest Measured Prior to the Outcome(S)?	7. Was the Timeframe Sufficient to See an Association Between Exposure and Outcome if It Existed?	8. Did the Study Examine Different Levels of the Exposure as Related to the Outcome?	9. Were the Exposure Measures Clearly Defined, Valid, Reliable, and Implemented Consistently?	10. Was the Exposure(S) Assessed More Than Once over Time?	11. Were the Outcome Measures Clearly Defined, Valid, Reliable, and Implemented Consistently?	12. Were Assessors Blinded to the Exposure Status of Participants?	13. Was Loss to Follow-Up After Baseline 20% Or Less?	14. Were Key Potential Confounding Variables Measured and Adjusted Statistically?	Total
[48]	+	+	+	+	+	+	+	+	−	−	−	−	+	+	fair
[49]	+	+	−	+	+	−	+	−	−	O	−	O	+	−	poor
[7]	+	+	+	+	+	+	+	+	−	O	+	O	+	+	good
[50]	+	+	+	+	+	+	+	O	−	−	+	O	+	+	good
[47]	+	+	+	+	+	+	+	−	−	−	+	O	+	−	fair
[51]	+	+	+	+	−	+	+	−	−	+	−	O	+	O	poor
[52]	+	−	−	+	+	+	+	+	−	+	−	O	+	+	fair
[53]	−	+	+	+	−	−	+	−	+	−	+	O	O	−	poor
[54]	+	+	+	+	−	+	+	−	−	−	−	O	+	−	poor
[55]	+	+	+	+	−	+	+	+	+	+	+	O	−	−	fair
[38]	+	+	−	+	−	+	+	O	+	−	+	O	O	+	good
[37]	+	+	−	+	−	+	+	+	+	−	+	O	+	+	good
[56]	+	+	+	+	−	+	+	+	+	−	+	O	−	+	good
[39]	+	+	O	+	−	+	+	+	−	−	+	O	−	−	poor
[40]	+	+	−	+	+	O	−	+	−	−	+	O	−	+	fair
[46]	−	−	+	+	−	+	+	−	+	−	+	O	−	−	poor
[57]	+	+	+	+	−	+	+	+	−	−	+	O	+	−	fair
[42]	+	+	−	+	−	+	+	+	+	−	+	O	−	+	fair
[41]	+	+	−	+	−	+	+	+	+	−	+	O	−	+	fair
[29]	+	+	+	+	−	+	+	+	+	−	+	O	+	+	good
[30]	+	+	+	+	−	+	+	+	+	−	+	O	+	+	good
[58]	+	+	+	+	−	+	+	+	+	−	+	O	O	+	good
[59]	−	−	−	+	−	+	+	+	+	−	+	O	+	+	fair
[60]	+	+	−	+	−	+	+	−	+	−	−	O	O	+	fair
[61]	−	+	−	+	−	+	+	−	−	−	−	O	+	−	poor
[62]	+	+	+	+	−	+	−	O	+	O	+	O	O	+	good
[31]	+	+	+	+	−	+	+	+	+	O	+	O	−	+	good
[63]	+	+	−	+	−	+	O	−	+	O	+	O	O	O	good
[64]	+	+	+	+	−	+	+	+	+	O	+	O	−	−	good
[65]	+	+	+	+	−	+	+	+	+	O	+	O	+	−	good
[66]	−	+	+	+	−	+	+	−	+	−	−	−	+	−	poor
[45]	+	+	+	+	−	+	+	−	+	O	+	O	+	−	fair
[67]	+	+	+	+	−	+	+	−	+	O	+	−	−	−	fair
[68]	+	+	+	+	−	+	+	−	+	O	+	−	O	+	good
[69]	+	+	O	+	+	+	+	+	+	O	+	−	O	−	good
[43]	+	+	+	+	−	+	+	−	+	O	+	O	+	−	good
[44]	+	+	+	+	−	+	+	+	+	O	+	O	+	−	good
[70]	+	+	−	+	−	+	−	+	+	−	−	O	O	−	poor
[71]	+	+	+	+	−	+	+	−	+	+	+	O	+	+	good
[32]	+	+	O	+	+	+	+	+	O	−	O	O	−	+	good
[72]	+	+	+	+	−	+	+	+	+	−	+	O	+	+	good
[73]	+	+	+	+	−	+	+	O	+	−	+	+	+	+	good
[74]	+	+	−	+	−	−	−	+	+	−	+	O	O	+	fair
[75]	+	+	+	−	−	+	−	O	+	−	+	O	O	−	fair
[76]	+	+	−	+	−	+	+	−	+	−	+	−	−	+	poor
[77]	−	−	−	+	−	+	+	−	−	−	−	O	O	−	poor
[78]	+	+	−	+	−	+	+	−	+	−	−	−	O	+	poor
[79]	+	+	−	+	−	+	+	−	+	−	−	O	O	−	poor
[80]	+	+	−	+	−	+	+	+	+	−	−	O	+	−	fair
[81]	−	+	−	−	−	+	−	−	+	−	+	−	+	−	poor
[82]	+	+	−	+	−	−	−	O	+	−	−	O	O	+	poor
[83]	−	−	−	−	−	−	−	−	−	−	−	O	O	−	poor
[84]	+	+	−	+	−	+	+	−	+	+	+	O	−	−	fair

Possible ratings were + (yes), − (no, not reported, could not be determined) and O (not applicable).

**Table 3:** Return to work percentages by time point.

Author (Year)	RTW Specified As	1 Week	2 Weeks	1 Month	1.5 Months	2 Months	3 Months	4.5 Months	6 Months	12 Months	24 Months	Not Specified/Various
Ackland (2013) [48]	Return to full duties	30.9%	43.2%	57.4%	71.0%		79.6%			89.5%		
Biering-Sorensen (2014) [50]	RTW								42.0%	50.6%	56.0%	
Borchgrevink (1996) [47]	Return to part-time and full-time work											72.5%
Buitenhuis (2009) [52]	Return to paid employment			79.4%					77.2%	76.0%		
Dufton (2006) [40]	RTW			46.6%								
Ettlin (1992) [46]	Return to partial and full employment						71.4%			100%	100%	
Geldman (2008) [57]	Return to usual work						50.0%		79.8%			
Gopinath (2015) * [42]	RTW									85.3%	82.3%	
Gopinath (2017) * [41]	RTW										82.0%	
Gray (2018) [29]	Sustained RTW											84.0%
Gun (2005) [59]	RTW									90.0%		
Holm (1999) [62]	Return to full work capacity											Year 1989: 63.0% Year 1994: 69.0%
Hours (2014) [31]	RTW									90.0%		
Kasch (2001) [64]	RTW									92.2%		
Kasch (2011) [65]	RTW									91.0%		
Kinzel (2006) [45]	RTW									36.0%		
Mankovsky-Arnold (2017) [69]	RTW					27.0%						
Nguyen (2019) [32]	RTW											Low risk: 91.0% High risk: 54.6%
O’Hara (2018) [72]	RTW										63.0%	
Pieske (2010) [73]	Able to work			90.1%					96.3%			
Prang (2015) [74]	RTW											74.0%
Rosenthal (1979) [77,85]	RTW											50.0%
Sarrami (2016) [78]	RTW										37.0%	
Schreiber (2009) [79]	Return to employment							74.2%				
Scuderi (2005) [80]	RTW											79.3%

Abbreviations: MSD, musculoskeletal disorders; RTW, return to work; WAD, whiplash-associated disorder. * Same cohort. Gopinath (2015) will be used for the analysis.

**Table 4:** Sick leave percentages reported as duration of sick leave or used sick leave at a certain time point.

Author (Year)	Sick Leave Specified As	Duration of Reported Sick Leave (E.G., Percentage of Participants that Reported Taking Sick Leave of the Specified Duration)			Reported Sick Leave Measured at Time Point (E.G., Percentage of Participants that Reportedly Used Sick Leave at a Certain Time Point)							Not Specified/Various
		<2 weeks	>2 weeks, <1 month	>1 month	Baseline	1 month	3 months	6 months	12 months	24 months	3 years	
Barbosa (2013) [49]	Time off work	20.0%	10.9%	20.0%								
Berecki-Gisolf (2013) [7]	Work disability days											27.0% of non-hospital group; 44.0% of 1–7 days hospital stay group; 29.0% of >1-week hospital group
Biering-Sorensen (2014) [50]	Sick listed							78.0%	47.0%	20.0%	8.0%	
Borchgrevink (1996) [47]	Registered for sick leave	14.0%	8.0%									
Brison (2000) [51]	Missing work	62.0%										
Buitenhuis (2009) [52]	Being on work disability					33.9%		18.9%	12.6%			
Bylund (1998) [54]	Sick leave									40.0%		
Carroll (2012) [55]	Off work					17.9%	10.5%	7.1%				
Dufton (2012) [39]	Off work											At discharge assessment: group 1 56.9%; Group 2 52.8%; Group 3 32.7%.
Herrström (2000) [60]	Sick leave			12.0%					32%			
Hildingsson (1990) [61]	Sick leave									14.0%		
Hours (2014) [31]	Sickness leave								52.6%			
Miettinen (2004) [43]	Sick leave	12.6%	14.8%	11.5%					39%			
Pieske (2010) [73]	Inability to work					9.9%	6.2%	3.7%				
Rebbeck (2006) [76]	Workdays off						24.4%	20.2%		16.3%		
Smed (1997) [81]	Sick leave	38.5%		15.4%		61.5%						
Virani (2001) [83]	Time off work											36.0%
Vos (2008) [84]	Sick leave				36.0%							

## Data Availability

Data sharing not applicable.

## Supplementary Materials

The following are available online at https://www.mdpi.com/article/10.3390/ijerph182111504/s1, SM1: Search strategy; SM2: Work outcomes of RTW, sick leave, work capacity, work ability and productivity loss; SM3: Pooled RTW percentages by time point and quality of evidence; SM4: Pooled sick leave percentages, reported as duration of sick leave or used sick leave at a certain time point; SM5: Sensitivity analysis RTW proportions; SM6: Sensitivity analysis sick leave proportions; SM7: Factors associated with work-related outcomes.
