# Supplementary material for: The Impact of Musculoskeletal Injuries Sustained in Road Traffic Crashes on Work-Related Outcomes: A Systematic Review
Source: Int J Environ Res Public Health. 2021 Nov 1;18(21):11504. doi: 10.3390/ijerph182111504 (PMC8582890; doi:10.3390/ijerph182111504)
Supplement: Supplementary file 1 [file ijerph-18-11504-s001.zip › ijerph-1395892-supplementary.pdf]

Supplementary Material 1.

PubMed Search Strategy

Work related search terms

#1 work [Title/abstract] OR job [Title/abstract] OR jobs [Title/abstract] OR occupation [Title/abstract] OR occupations [Title/abstract] OR occupational [Title/abstract] OR vocation [Title/abstract] OR vocations [Title/abstract] OR vocational [Title/abstract] OR employee [Title/abstract] OR employees [Title/abstract] OR employer [Title/abstract] OR employers [Title/abstract] OR worker [Title/abstract] OR workers [Title/abstract] OR workplace [Title/ abstract] OR employability [Title/abstract] OR unemployment [Title/ abstract] OR employment [Title/abstract] OR absentee [Title/ abstract] OR "sick listed"[Title/abstract] OR sicklisted [Title/abstract] OR "sick leave"[Title/abstract] OR "sick absence"[Title/abstract] OR "sickness leave"[Title/abstract] OR "sickness absence"[Title/abstract] OR "sick days"[Title/abstract] OR "sick day"[Title/abstract] OR "illness day"[Title/abstract] OR "illness days"[Title/abstract] OR absenteeism [Title/abstract] OR presenteeism [Title/abstract] OR "workday loss"[Title/abstract] OR "workdays lost"[Title/abstract] OR workloss [Title/abstract] OR "disability evaluation"[Title/abstract] OR "disability prevention"[Title/abstract] OR "disability leave"[Title/abstract] OR "functional capacity evaluation"[Title/abstract] OR productivity [Title/abstract]

#2 work [MeSH] OR employment [MeSH] OR "sick leave"[MeSH] OR "work capacity evaluation"[MeSH]

#3 1OR2

Road traffic crash-related search terms

#4 (Car [Title/abstract] OR Cars [Title/abstract] OR Truck [Title/abstract] OR Trucks [Title/abstract] OR Automobile [Title/abstract] OR Automobiles [Title/abstract] OR Vehicle [Title/abstract] OR Vehicles [Title/abstract] OR Vehicular [Title/abstract] OR Cycle [Title/abstract] OR Cycles [Title/abstract] OR Cyclist [Title/abstract] OR Cyclists [Title/abstract] OR Cycling [Title/abstract] OR Bicycle [Title/abstract] OR bicycles [Title/abstract] OR Pedestrian [Title/abstract] OR Pedestrians [Title/abstract] OR Passenger [Title/abstract] OR Passengers [Title/abstract] OR Driver [Title/abstract] OR Drivers [Title/abstract] OR motor [Title/abstract] OR motorbike [Title/ abstract] OR motorbikes [Title/abstract] OR motorbiker [Title/ abstract] OR motorbikers [Title/abstract] OR motorcar [Title/ abstract] OR motorcars [Title/abstract] OR motorcycle [Title/ abstract] OR motorcycles [Title/abstract] OR motorcycling [Title/ abstract] OR motorcyclist [Title/abstract] OR motorcyclists [Title/ abstract] OR motorhome [Title/abstract] OR motorhomes [Title/ abstract] OR motorist [Title/abstract] OR motorists [Title/abstract] OR motorvehicle [Title/abstract] OR motorvehicles [Title/abstract] OR transport [Title/abstract] OR transportation [Title/abstract] OR traffic [Title/abstract] OR road [Title/abstract] OR roads [Title/ abstract] OR roadside [Title/abstract] OR roadsides [Title/abstract]) AND (accident [Title/abstract] OR accidents [Title/abstract] OR collision [Title/abstract] OR collisions [Title/abstract] OR crash [Title/ abstract] OR crashes [Title/abstract] OR crashed [Title/abstract] OR smash [Title/abstract] OR smashes [Title/abstract] OR smashed [Title/abstract])

#5 “Accidents, Traffic”[MeSH]

#6 “Whiplash injuries”[MeSH] OR whiplash [Title/abstract]

#7 (road [Title/abstract] OR traffic [Title/abstract]) AND
(injury [Title/abstract] OR injuries [Title/abstract] OR trauma [Title/abstract])

#8 4OR5OR6OR7

Study type

#9 Crosssectional [Title/abstract] OR cross-sectional [Title/abstract] OR “cross sectional”[Title/abstract] OR observational [Title/abstract] OR casecontrol [Title/abstract] OR case-control [Title/abstract] OR “case control”[Title/abstract] OR cohort [Title/abstract] OR longitudinal [Title/abstract] OR ((prospective [Title/abstract] OR retrospective [Title/abstract]) AND (cohort [Title/abstract] OR study [Title/abstract] OR observational [Title/abstract] OR longitudinal [Title/abstract]))

Musculoskeletal injury

#10 Musculoskeletal [Title/abstract] OR myofascial [Title/abstract] OR arthralgia [Title/abstract] OR arthropathy [Title/abstract] OR arthritis [Title/abstract] OR arthritic [Title/abstract] OR myalgia [Title/ abstract] OR backache [Title/abstract] OR whiplash [Title/abstract]

#11 “Musculoskeletal diseases”[MeSH] OR “Musculoskeletal system”[MeSH] OR “Wounds and injuries”[MeSH] OR “Wounds, Nonpenetrating”[MeSH] OR “Musculoskeletal pain”[MeSH] OR “Back pain”[MeSH] OR “Myofascial pain syndromes”[MeSH] OR Arthralgia [MeSH] OR “Neck pain”[MeSH] OR “brachial plexus neuropathies”[MeSH] OR “Fractures, Bone”[MeSH] OR “Joint Dislocations”[MeSH] OR “Soft tissue injuries”[MeSH] OR “Multiple trauma”[MeSH] OR Orthopedics [MeSH] OR “Orthopedic Procedures”[MeSH]

#12 (Fracture [Title/abstract] OR Fractures [Title/abstract] OR Fractured [Title/abstract] OR sprain [Title/abstract] OR sprains [Title/abstract] sprained [Title/abstract] OR dislocation [Title/abstract] OR dislocations [Title/abstract] OR dislocated [Title/abstract] OR injury [Title/abstract] OR injuries [Title/abstract] OR injured [Title/abstract] OR contusion [Title/abstract] OR contusions [Title/abstract] OR oedema [Title/abstract] OR edema [Title/abstract] OR trauma [Title/ abstract] OR multitrauma [Title/abstract] OR multi-trauma [Title/ abstract] OR “multi trauma”[Title/abstract] OR orthopaedic [Title/ abstract] OR orthopaedics [Title/abstract] OR orthopedic [Title/ abstract] OR orthopedics [Title/abstract] OR surgery [Title/abstract] OR ache [Title/abstract] OR pain [Title/abstract]) AND (Arm [Title/ abstract] OR arms [Title/abstract] OR “upper limb”[Title/abstract] OR “upper limbs”[Title/abstract] OR “upper extremity”[Title/abstract] OR “upper extremities”[Title/abstract] OR leg [Title/abstract] OR legs [Title/abstract] OR “lower limb”[Title/abstract] OR “lower limbs” [Title/abstract] OR “lower extremity”[Title/abstract] OR “lower extremities”[Title/abstract] OR shoulder [Title/abstract] OR shoulders [Title/abstract] OR “rotator cuff”[Title/abstract] OR humerus [Title/ abstract] OR humeral [Title/abstract] OR elbow [Title/abstract] OR elbows [Title/abstract] OR forearm [Title/abstract] OR forearms [Title/abstract] OR radius [Title/abstract] OR radial

[Title/abstract] OR ulna [Title/abstract] OR ulnar [Title/abstract] OR wrist [Title/abstract] OR wrists [Title/abstract] OR hand [Title/abstract] OR hands [Title/ abstract] OR finger [Title/abstract] OR fingers [Title/abstract] OR thumb [Title/abstract] OR thumbs [Title/abstract] OR spine [Title/ abstract] OR spinal [Title/abstract] OR cervical [Title/abstract] OR thoracic [Title/abstract] OR thorax [Title/abstract] OR lumbar [Title/ abstract] OR sacral [Title/abstract] OR sacrum [Title/abstract] OR neck [Title/abstract] OR chest [Title/abstract] OR back [Title/ abstract] OR pelvis [Title/abstract] OR pelvic [Title/abstract] OR hip [Title/abstract] OR hips [Title/abstract] OR knee [Title/abstract] OR knees [Title/abstract] OR ankle [Title/abstract] OR ankles [Title/ abstract] OR thigh [Title/abstract] OR thighs [Title/abstract] OR femur [Title/abstract] OR femoral [Title/abstract] OR tibia [Title/ abstract] OR tibial [Title/abstract] OR fibula [Title/abstract] OR fibular [Title/abstract] OR shin [Title/abstract] OR shins [Title/abstract] OR foot [Title/abstract] OR feet [Title/abstract] OR toe [Title/abstract] OR toes [Title/abstract] OR metatarsal [Title/abstract] OR metatarsals [Title/abstract] OR mandibular [Title/abstract] OR maxillofacial [Title/ abstract] OR ligament [Title/abstract] OR ligaments [Title/abstract] OR muscle [Title/abstract] OR muscles [Title/abstract] OR “soft tissue” [Title/abstract] OR tendon [Title/abstract] OR tendons [Title/abstract])

#13 10OR11OR12 Combined

Final Search Term

#14 3AND8AND9AND13

#15 Limit to English

<u>EMBASE Search Strategy</u>

Work related search terms

#1 (work:ab,ti OR job:ab,ti OR jobs:ab,ti OR occupation:ab,ti OR occupations:ab,ti OR occupational:ab,ti OR vocation:ab,ti OR vocations:ab,ti OR vocational:ab,ti OR employee:ab,ti OR employees:ab,ti OR employer:ab,ti OR employers:ab,ti OR worker:ab,ti OR workers:ab,ti OR workplace:ab,ti OR employability:ab,ti OR unemployment:ab,ti OR employment:ab,ti OR absentee:ab,ti OR ‘sick listed’:ab,ti OR sicklisted:ab,ti OR ‘sick leave’:ab,ti OR ‘sick absence’:ab,ti OR ‘sickness leave’:ab,ti OR ‘sickness absence’:ab,ti OR ‘sick days’:ab,ti OR ‘sick day’:ab,ti OR ‘illness day’:ab,ti OR ‘illness days’:ab,ti OR absenteeism:ab,ti OR presenteeism:ab,ti OR ‘workday loss’:ab,ti OR ‘workdays lost’:ab,ti OR workloss:ab,ti OR ‘disability evaluation’:ab,ti OR ‘disability prevention’:ab,ti OR ‘disability leave’:ab,ti OR ‘medical leave’:ab,ti OR ‘functional capacity evaluation’:ab,ti OR productivity:ab,ti) AND [embase]/lim

#2 (Work/exp OR employment/exp OR ‘vocational rehabilitation’/exp) AND [embase]/lim

#3 1 OR 2

Road traffic crash-related search terms

#4 ((Car:ab,ti OR Cars:ab,ti OR Truck:ab,ti OR Trucks:ab,ti OR Automobile:ab,ti OR Automobiles:ab,ti OR Vehicle:ab,ti OR Vehicles:ab,ti OR Vehicular:ab,ti OR Cycle:ab,ti OR Cycles:ab,ti OR Cyclist:ab,ti OR Cyclists:ab,ti OR Cycling:ab,ti OR Bicycle:ab,ti OR bicycles:ab,ti OR Pedestrian:ab,ti OR Pedestrians:ab,ti OR Passenger:ab,ti OR Passengers:ab,ti OR Driver:ab,ti OR Drivers:ab,ti OR motor:ab,ti OR motorbike:ab,ti OR motorbikes:ab,ti OR motorbiker:ab,ti OR motorbikers:ab,ti OR motorcar:ab,ti OR motorcars:ab,ti OR motorcycle:ab,ti OR motorcycles:ab,ti OR motorcycling:ab,ti OR motorcyclist:ab,ti OR motorcyclists:ab,ti OR motorhome:ab,ti OR motorhomes:ab,ti OR motorist:ab,ti OR motorists:ab,ti OR motorvehicle:ab,ti OR motorvehicles:ab,ti OR transport:ab,ti OR transportation:ab,ti OR traffic:ab,ti OR road:ab,ti OR roads:ab,ti OR roadside:ab,ti OR roadsides:ab,ti) AND (accident:ab,ti OR accidents:ab,ti OR collision:ab,ti OR collisions:ab,ti OR crash:ab,ti OR crashes:ab,ti OR crashed:ab,ti OR smash:ab,ti OR smashes:ab,ti OR smashed:ab,ti)) AND [embase]/lim

#5 ‘traffic accident’/exp AND [embase]/lim

#6 (‘whiplash injury’/exp OR whiplash:ab,ti) AND [embase]/lim

#7 (road:ab,ti OR traffic:ab,ti) AND (injury:ab,ti OR injuries:ab,ti OR trauma:ab,ti) AND [embase]/lim

#8 4 OR 5 OR 6 OR 7

Study type

#9 (Crosssectional:ab,ti OR ‘cross sectional’:ab,ti OR observational:ab,ti OR casecontrol:ab,ti OR ‘case-control’:ab,ti OR cohort:ab,ti OR longitudinal:ab,ti OR ((prospective:ab,ti OR retrospective:ab,ti) AND (cohort:ab,ti OR study:ab,ti OR observational:ab,ti OR longitundinal:ab,ti))) AND [embase]/lim

Musculoskeletal injury

#10 (Musculoskeletal:ab,ti OR myofascial:ab,ti OR arthralgia:ab,ti OR arthropathy:ab,ti OR arthritis:ab,ti OR arthritic:ab,ti OR myalgia:ab,ti OR backache:ab,ti OR whiplash:ab,ti) AND [embase]/lim

#11 (’Musculoskeletal disease’/exp OR Injury/exp OR ‘Musculoskeletal pain’/exp OR myalgia/exp OR Arthralgia/exp OR ‘brachial plexus neuropathies’/exp) AND [embase]/lim

12 ((Fracture:ab,ti OR Fractures:ab,ti OR Fractured:ab,ti OR sprain:ab,ti OR sprains:ab,ti sprained:ab,ti OR dislocation:ab,ti OR dislocations:ab,ti OR dislocated:ab,ti OR injury:ab,ti OR injuries:ab,ti OR injured:ab,ti OR contusion:ab,ti OR contusions:ab,ti OR oedema:ab,ti OR edema:ab,ti OR trauma:ab,ti OR multitrauma:ab,ti OR multi-trauma:ab,ti OR “multi trauma”:ab,ti OR orthopaedic:ab,ti OR orthopaedics:ab,ti OR orthopedic:ab,ti OR orthopedics:ab,ti OR surgery:ab,ti OR ache:ab,ti OR pain:ab,ti) AND (Arm:ab,ti OR arms:ab,ti OR “upper limb”:ab,ti OR “upper limbs”:ab,ti OR “upper extremity”:ab,ti OR “upper extremities”:ab,ti OR leg:ab,ti OR legs:ab,ti OR “lower limb”:ab,ti OR “lower limbs”:ab,ti OR “lower extremity”:ab,ti OR “lower extremities”:ab,ti OR shoulder:ab,ti OR

shoulders:ab,ti OR “rotator cuff”:ab,ti OR humerus:ab,ti OR humeral:ab,ti OR elbow:ab,ti OR elbows:ab,ti OR forearm:ab,ti OR forearms:ab,ti OR radius:ab,ti OR radial:ab,ti OR ulna:ab,ti OR ulnar:ab,ti OR wrist:ab,ti OR wrists:ab,ti OR hand:ab,ti OR hands:ab,ti OR finger:ab,ti OR fingers:ab,ti OR thumb:ab,ti OR thumbs:ab,ti OR spine:ab,ti OR spinal:ab,ti OR cervical:ab,ti OR thoracic:ab,ti OR thorax:ab,ti OR lumbar:ab,ti OR sacral:ab,ti OR sacrum:ab,ti OR neck:ab,ti OR chest:ab,ti OR back:ab,ti OR pelvis:ab,ti OR pelvic:ab,ti OR hip:ab,ti OR hips:ab,ti OR knee:ab,ti OR knees:ab,ti OR ankle:ab,ti OR ankles:ab,ti OR thigh:ab,ti OR thighs:ab,ti OR femur:ab,ti OR femoral:ab,ti OR tibia:ab,ti OR tibial:ab,ti OR fibula:ab,ti OR fibular:ab,ti OR shin:ab,ti OR shins:ab,ti OR foot:ab,ti OR feet:ab,ti OR toe:ab,ti OR toes:ab,ti OR metatarsal:ab,ti OR metatarsals:ab,ti OR mandibular:ab,ti OR maxillofacial:ab,ti OR ligament:ab,ti OR ligaments:ab,ti OR muscle:ab,ti OR muscles:ab,ti OR “soft tissue”:ab,ti OR tendon:ab,ti OR tendons:ab,ti)) AND [embase]/lim

#13 10 OR 11 OR 12

Final Search Term

#14 3 AND 8 AND 9 AND 13

#15 Limit to English

<u>CINAHL Search Strategy</u>

Work related search terms

#1 TI (work OR job OR jobs OR occupation OR occupations OR occupational OR vocation OR vocations OR vocational OR employee OR employees OR employer OR employers OR worker OR workers OR workplace OR employability OR unemployment OR employment OR absentee OR “sick listed” OR sicklisted OR “sick leave” OR “sick absence” OR “sickness leave” OR “sickness absence” OR “sick days” OR “sick day” OR “illness day” OR “illness days” OR absenteeism OR presenteeism OR “workday loss” OR “workdays lost” OR workloss OR “disability evaluation” OR “disability prevention” OR “disability leave” OR “functional capacity evaluation” OR productivity) OR

AB (work OR job OR jobs OR occupation OR occupations OR occupational OR vocation OR vocations OR vocational OR employee OR employees OR employer OR employers OR worker OR workers OR workplace OR employability OR unemployment OR employment OR absentee OR “sick listed” OR sicklisted OR “sick leave” OR “sick absence” OR “sickness leave” OR “sickness absence” OR “sick days” OR “sick day” OR “illness day” OR “illness days” OR absenteeism OR presenteeism OR “workday loss” OR “workdays lost” OR workloss OR “disability evaluation” OR “disability prevention” OR “disability leave” OR “functional capacity evaluation” OR productivity)

#2 MH (Work OR Job Re-Entry OR Job Performance OR Employment OR Unemployment OR Rehabilitation, Vocational OR Sick Leave OR Work Capacity Evaluation)

#3 1 OR 2

Road traffic crash-related search terms

#4 TI ((Car OR Cars OR Truck OR Trucks OR Automobile OR Automobiles OR Vehicle OR Vehicles OR Vehicular OR Cycle OR Cycles OR Cyclist OR Cyclists OR Cycling OR Bicycle OR bicycles OR Pedestrian OR Pedestrians OR Passenger OR Passengers OR Driver OR Drivers OR motor OR motorbike OR motorbikes OR motorbiker OR motorbikers OR motorcar OR motorcars OR motorcycle OR motorcycles OR motorcycling OR motorcyclist OR motorcyclists OR motorhome OR motorhomes OR motorist OR motorists OR motorvehicle OR motorvehicles OR transport OR transportation OR traffic OR road OR roads OR roadside OR roadsides) AND (accident OR accidents OR collision OR collisions OR crash OR crashes OR crashed OR smash OR smashes OR smashed))

OR AB ((Car OR Cars OR Truck OR Trucks OR Automobile OR Automobiles OR Vehicle OR Vehicles OR Vehicular OR Cycle OR Cycles OR Cyclist OR Cyclists OR Cycling OR Bicycle OR bicycles OR Pedestrian OR Pedestrians OR Passenger OR Passengers OR Driver OR Drivers OR motor OR motorbike OR motorbikes OR motorbiker OR motorbikers OR motorcar OR motorcars OR motorcycle OR motorcycles OR motorcycling OR motorcyclist OR motorcyclists OR motorhome OR motorhomes OR motorist OR motorists OR motorvehicle OR motorvehicles OR transport OR transportation OR traffic OR road OR roads OR roadside OR roadsides) AND (accident OR accidents OR collision OR collisions OR crash OR crashes OR crashed OR smash OR smashes OR smashed))

#5 MH “Accidents, Traffic”

#6 MH “Whiplash injuries” OR TI Whiplash OR AB Whiplash

#7 (TI (road OR traffic) AND TI (injury OR injuries OR trauma)) OR (AB (road OR traffic) AND AB (injury OR injuries OR trauma))

#8 4 OR 5 OR 6 OR 7

Study type

#9 TI (Crosssectional OR “cross-sectional” OR observational OR casecontrol OR “case-control” OR cohort OR longitudinal OR ((prospective OR retrospective) AND (cohort OR study OR observational OR longitudinal))) OR AB (Crosssectional OR “cross-sectional” OR

observational OR casecontrol OR “case-control” OR cohort OR longitudinal OR ((prospective OR retrospective) AND (cohort OR study OR observational OR longitudinal)))

Musculoskeletal injury

#10 TI (Musculoskeletal OR myofascial OR arthralgia OR arthropathy OR arthritis OR arthritic OR myalgia OR backache OR whiplash) OR AB (Musculoskeletal OR myofascial OR arthralgia OR arthropathy OR arthritis OR arthritic OR myalgia OR backache OR whiplash)

#11 MH (“musculoskeletal diseases” OR “musculoskeletal system” OR “wounds and injuries” OR “Back Pain” OR “Muscle Pain” OR “Neck Pain” OR Arthralgia OR “Shoulder Pain” OR “Myofascial Pain Syndromes” OR “Brachial Plexus Neuropathies”)

#12 TI ((Fracture OR Fractures OR Fractured OR sprain OR sprains sprained OR dislocation OR dislocations OR dislocated OR injury OR injuries OR injured OR contusion OR contusions OR oedema OR edema OR trauma OR multitrauma OR multi-trauma OR “multi trauma” OR orthopaedic OR orthopaedics OR orthopedic OR orthopedics OR surgery OR ache OR pain) AND (Arm OR arms OR “upper limb” OR “upper limbs” OR “upper extremity” OR “upper extremities” OR leg OR legs OR “lower limb” OR “lower limbs” OR “lower extremity” OR “lower extremities” OR shoulder OR shoulders OR “rotator cuff” OR humerus OR humeral OR elbow OR elbows OR forearm OR forearms OR radius OR radial OR ulna OR ulnar OR wrist OR wrists OR hand OR hands OR finger OR fingers OR thumb OR thumbs OR spine OR spinal OR cervical OR thoracic OR thorax OR lumbar OR sacral OR sacrum OR neck OR chest OR back OR pelvis OR pelvic OR hip OR hips OR knee OR knees OR ankle OR ankles OR thigh OR thighs OR femur OR femoral OR tibia OR tibial OR fibula OR fibular OR shin OR shins OR foot OR feet OR toe OR toes OR metatarsal OR metatarsals OR mandibular OR maxillofacial OR ligament OR ligaments OR muscle OR muscles OR “soft tissue” OR tendon OR tendons)) OR AB ((Fracture OR Fractures OR Fractured OR sprain OR sprains sprained OR dislocation OR dislocations OR dislocated OR injury OR injuries OR injured OR contusion OR contusions OR oedema OR edema OR trauma OR multitrauma OR multi-trauma OR “multi trauma” OR orthopaedic OR orthopaedics OR orthopedic OR orthopedics OR surgery OR ache OR pain) AND (Arm OR arms OR “upper limb” OR “upper limbs” OR “upper extremity” OR “upper extremities” OR leg OR legs OR “lower limb” OR “lower limbs” OR “lower extremity” OR “lower extremities” OR shoulder OR shoulders OR “rotator cuff” OR humerus OR humeral OR elbow OR elbows OR forearm OR forearms OR radius OR radial OR ulna OR ulnar OR wrist OR wrists OR hand OR hands OR finger OR fingers OR thumb OR thumbs OR spine OR spinal OR cervical OR thoracic OR thorax OR lumbar OR sacral OR sacrum OR neck OR chest OR back OR pelvis OR pelvic OR hip OR hips OR knee OR knees OR ankle OR ankles OR thigh OR thighs OR femur OR femoral OR tibia OR tibial OR fibula OR fibular OR shin OR shins OR foot OR feet OR toe OR toes OR metatarsal OR metatarsals OR mandibular OR maxillofacial OR ligament OR ligaments OR muscle OR muscles OR “soft tissue” OR tendon OR tendons))

#13 10 OR 11 OR 12

Final Search Term

#14	3 AND 8 AND 9 AND 13

#15	Limit to English

Web of Science core collection Search Strategy

Work related search terms

#1 TS=(work OR job OR jobs OR occupation OR occupations OR occupational OR vocation OR vocations OR vocational OR employee OR employees OR employer OR employers OR worker OR workers OR workplace OR employability OR unemployment OR employment OR absentee OR "sick listed" OR sicklisted OR "sick leave" OR "sick absence" OR "sickness leave" OR "sickness absence" OR "sick days" OR "sick day" OR absenteeism OR presenteeism OR "workday loss" OR "workdays lost" OR workloss OR "disability evaluation" OR "disability prevention" OR "functional capacity evaluation" OR productivity OR "disability leave" OR "medical leave" OR "illness day" OR "illness days")

Road traffic crash-related search terms

#2	TS=(Car OR Cars OR Truck OR Trucks OR Automobile OR Automobiles OR Vehicle OR Vehicles OR Vehicular OR Cycle OR Cycles OR Cyclist OR Cyclists OR Cycling OR Bicycle OR bicycles OR Pedestrian OR Pedestrians OR Passenger OR Passengers OR Driver OR Drivers OR motor OR motorbike OR motorbikes OR motorbiker OR motorbikers OR motorcar OR motorcars OR motorcycle OR motorcycles OR motorcycling OR motorcyclist OR motorcyclists OR motorhome OR motorhomes OR motorist OR motorists OR motorvehicle OR motorvehicles OR transport OR transportation OR traffic OR road OR roads OR roadside OR roadsides) AND TS=(accident OR accidents OR collision OR collisions OR crash OR crashes OR crashed OR smash OR smashes OR smashed)

#3	TS=(road OR traffic) AND TS=(injury OR injuries OR trauma)

#4	TS=whiplash

#5	2 OR 3 OR 4

Study type

#6 TS=(Crosssectional OR "cross-sectional" OR "cross sectional" OR observational OR casecontrol OR "case-control" OR "case control" OR cohort OR longitudinal OR ((prospective OR retrospective) AND (cohort OR study OR observational OR longitudinal)))

Musculoskeletal injury

#7 TS=(Musculoskeletal OR myofascial OR arthralgia OR arthropathy OR arthritis OR arthritic OR myalgia OR backache OR whiplash)

#8 TS=(Fracture OR Fractures OR Fractured OR sprain OR sprains sprained OR dislocation OR dislocations OR dislocated OR injury OR injuries OR injured OR contusion OR contusions OR oedema OR edema OR trauma OR multitrauma OR multi-trauma OR “multi trauma” OR orthopaedic OR orthopaedics OR orthopedic OR orthopedics OR surgery OR ache OR pain) AND TS=(Arm OR arms OR “upper limb” OR “upper limbs” OR “upper extremity” OR “upper extremities” OR leg OR legs OR “lower limb” OR “lower limbs” OR “lower extremity” OR “lower extremities” OR shoulder OR shoulders OR “rotator cuff” OR humerus OR humeral OR elbow OR elbows OR forearm OR forearms OR radius OR radial OR ulna OR ulnar OR wrist OR wrists OR hand OR hands OR finger OR fingers OR thumb OR thumbs OR spine OR spinal OR cervical OR thoracic OR thorax OR lumbar OR sacral OR sacrum OR neck OR chest OR back OR pelvis OR pelvic OR hip OR hips OR knee OR knees OR ankle OR ankles OR thigh OR thighs OR femur OR femoral OR tibia OR tibial OR fibula OR fibular OR shin OR shins OR foot OR feet OR toe OR toes OR metatarsal OR metatarsals OR mandibular OR maxillofacial OR ligament OR ligaments OR muscle OR muscles OR “soft tissue” OR tendon OR tendons)

#9 7 OR 8

Final Search Term

#10 1 AND 5 AND 6 AND 9

#11 Limit to English

Australian Transport Index (ATRI) Search Strategy

Work related search terms

#1 SUBJECT:work OR SUBJECT:job OR SUBJECT:jobs OR SUBJECT:occupation OR SUBJECT:occupations OR SUBJECT:occupational OR SUBJECT:vocation OR SUBJECT:vocations OR SUBJECT:vocational OR SUBJECT:employee OR SUBJECT:employees OR SUBJECT:employer OR SUBJECT:employers OR SUBJECT:worker OR SUBJECT:workers OR SUBJECT:workplace OR SUBJECT:employability OR SUBJECT:unemployment OR SUBJECT:employment OR SUBJECT:absentee OR SUBJECT:sick OR SUBJECT:absence OR SUBJECT:absenteeism OR SUBJECT:presenteeism OR SUBJECT:workloss OR SUBJECT:productivity

Road traffic crash-related search terms

#2 ((SUBJECT:Car OR SUBJECT:Cars OR SUBJECT:Truck OR SUBJECT:Trucks OR SUBJECT:Automobile OR SUBJECT:Automobiles OR SUBJECT:Vehicle OR SUBJECT:Vehicles OR SUBJECT:Vehicular OR SUBJECT:Cycle OR SUBJECT:Cycles OR SUBJECT:Cyclist OR SUBJECT:Cyclists OR SUBJECT:Cycling OR SUBJECT:Bicycle OR SUBJECT:bicycles OR SUBJECT:Pedestrian OR SUBJECT:Pedestrians OR SUBJECT:Passenger OR SUBJECT:Passengers OR SUBJECT:Driver OR SUBJECT:Drivers OR SUBJECT:motor OR SUBJECT:motorbike OR SUBJECT:motorbikes OR SUBJECT:motorbiker OR SUBJECT:motorbikers OR SUBJECT:motorcar OR SUBJECT:motorcars OR SUBJECT:motorcycle OR SUBJECT:motorcycles OR SUBJECT:motorcycling OR SUBJECT:motorcyclist OR SUBJECT:motorcyclists OR SUBJECT:motorhome OR SUBJECT:motorhomes OR SUBJECT:motorist OR SUBJECT:motorists OR SUBJECT:motorvehicle OR SUBJECT:motorvehicles OR SUBJECT:transport OR SUBJECT:transportation OR SUBJECT:traffic OR SUBJECT:road OR SUBJECT:roads OR SUBJECT:roadside OR SUBJECT:roadsides) AND (SUBJECT:accident OR SUBJECT:accidents OR SUBJECT:collision OR SUBJECT:collisions OR SUBJECT:crash OR SUBJECT:crashes OR SUBJECT:crashed OR SUBJECT:smash OR SUBJECT:smashes OR SUBJECT:smashed)) OR ((SUBJECT:road OR SUBJECT:traffic) AND (SUBJECT:injury OR SUBJECT:injuries OR SUBJECT:trauma)) OR (SUBJECT:whiplash)

Musculoskeletal injury

#3 (SUBJECT:Muscle OR SUBJECT:muscles OR SUBJECT:musculoskeletal OR SUBJECT:muscular OR SUBJECT:ligament OR SUBJECT:dislocation OR SUBJECT:injury OR SUBJECT:pain OR SUBJECT:sprain OR SUBJECT:strain OR SUBJECT:fracture OR SUBJECT:whiplash)

Final Search Term

#4 1 AND 2 AND 3

<u>PsychINFO Search Strategy</u>

Work related search terms

#1 work OR job OR jobs OR occupation OR occupations OR occupational OR vocation OR vocations OR vocational OR employee OR employees OR employer OR employers OR worker OR workers OR workplace OR employability OR unemployment OR employment OR absentee OR “sick listed” OR sicklisted OR “sick leave” OR “sick absence” OR “sickness leave” OR “sickness absence” OR “sick days” OR “sick day” OR “illness day” OR “illness days” OR absenteeism OR presenteeism OR “workday loss” OR “workdays lost” OR workloss OR “disability evaluation” OR “disability prevention” OR “disability leave” OR “functional capacity evaluation” OR productivity

Road traffic crash-related search terms

#2 (Car OR Cars OR Truck OR Trucks OR Automobile OR Automobiles OR Vehicle OR Vehicles OR Vehicular OR Cycle OR Cycles OR Cyclist OR Cyclists OR Cycling OR Bicycle OR bicycles OR Pedestrian OR Pedestrians OR Passenger OR Passengers OR Driver OR Drivers OR motor OR motorbike OR motorbikes OR motorbiker OR motorbikers OR motorcar OR motorcars OR motorcycle OR motorcycles OR motorcycling OR motorcyclist OR motorcyclists OR motorhome OR motorhomes OR motorist OR motorists OR motorvehicle OR motorvehicles OR transport OR transportation OR traffic OR road OR roads OR roadside OR roadsides) AND (accident OR accidents OR collision OR collisions OR crash OR crashes OR crashed OR smash OR smashes OR smashed)

#3 (road OR traffic) AND (injury OR injuries OR trauma)

#4 Whiplash

#5 2 OR 3 OR 4

Study type

#6 Crosssectional OR cross-sectional OR "cross sectional" OR observational OR casecontrol OR case-control OR "case control" OR cohort OR longitudinal OR ((prospective OR retrospective) AND (cohort OR study OR observational OR longitudinal))

Musculoskeletal injury

#7 Musculoskeletal OR myofascial OR arthralgia OR arthropathy OR arthritis OR arthritic OR myalgia OR backache OR whiplash

#8 (Fracture OR Fractures OR Fractured OR sprain OR sprains sprained OR dislocation OR dislocations OR dislocated OR injury OR injuries OR injured OR contusion OR contusions OR oedema OR edema OR trauma OR multitrauma OR multi-trauma OR "multi trauma" OR orthopaedic OR orthopaedics OR orthopedic OR orthopedics OR surgery OR ache OR pain) AND (Arm OR arms OR "upper limb" OR "upper limbs" OR "upper extremity" OR "upper extremities" OR leg OR legs OR "lower limb" OR "lower limbs" OR "lower extremity" OR "lower extremities" OR shoulder OR shoulders OR "rotator cuff" OR humerus OR humeral OR elbow OR elbows OR forearm OR forearms OR radius OR radial OR ulna OR ulnar OR wrist OR wrists OR hand OR hands OR finger OR fingers OR thumb OR thumbs OR spine OR spinal OR cervical OR thoracic OR thorax OR lumbar OR sacral OR sacrum OR neck OR chest OR back OR pelvis OR pelvic OR hip OR hips OR knee OR knees OR ankle OR ankles OR thigh OR thighs OR femur OR femoral OR tibia OR tibial OR fibula OR fibular OR shin OR shins OR foot OR feet OR toe OR toes OR metatarsal OR metatarsals OR mandibular OR maxillofacial OR ligament OR ligaments OR muscle OR muscles OR "soft tissue" OR tendon OR tendons)

#9 7 OR 8

Final Search Term

#10 1 AND 5 AND 6 AND 9

Supplementary Material 2

Table 1. Work Outcomes of return to work (RTW), sick leave, work capacity, work ability and productivity loss.

ID		Work Outcomes				
		**RTW**	**Sick leave**	**Work capacity**	**Work ability**	**Productivity loss**
54	Ackland (2013)	Return to full duties within 1 week n=50 (30.9%); 2 weeks n=70 (43.2%); 4 weeks n=93 (57.4%); 6 weeks n=115 (71.0%); 12 weeks n=129 (79.6%)<br><br>Not returned to work at 12m n=17 (10.5%).		Period of modified work prior to full duties 1-2 weeks n=44 (43.8%); 2-4 weeks n=16 (12.5%); 4-6 weeks n=11 (18.8%); 6-12 weeks n=4 (2.5%); >12 weeks n=20 (12.3%).	Patient-reported rationale for delay in return to full duties: neck injury or neck pain n=81 (50.0%); other traumatic injury (minor thoracolumbar or minor limb fracture) n=19 (11.7%); psychological issues n=12 (7.4%). Patient-reported rationale for not having returned to work at 12m: neck injury or pain n=7 (41.2%); other traumatic injury (minor thoracolumbar or minor limb fracture) n=5 (29.4%); psychological issues n=4 (28.6%); social issues n=1 (5.9%).	
121	Barbosa (2014)		Time off work non-facial injury: <24h n=13 (92.9%); 48h to 1week n=31 (93.9%); 2-3week n=17 (94.4%); >1month n=24 (72.7%).<br>Time off work facial injury: <24h n=1 (7.1%); 48h-1week n=2 (6. %); 2-3 week n=1 (5.6%); >1month n=9 (27.3%).			Absenteeism non-facial injury: Yes n=85 (86.7% of all people – non-facial and facial injuries); No n=65 (97% of all people)<br>Absenteeism facial-injury Yes n=13 (13.3% of all people); No n=2 (3.0% of all people)
151	Berecki-Gisolf (2013)		Compensated days off work: no hospital stay group 27%; 1-7 days hospital group 44%; >1-week hospital group 29%. Of those with compensated time off work, 32% had work disability over 6 months.			
162	Biering-Sorensen (2014)	WAD group: n=19 (18%) RTW at 26 weeks; n=35 (34%) at 52 weeks; n=46 (44%) at 2 years.<br>MSD group: n=1,369 (43%) RTW at 26 weeks; n=1,638 (51%) at 52 weeks; n=1,807 (57%) at 2 years.<br>Total RTW at 26 weeks n=1388/3308 (42%) ; at 52 weeks n=1673/3308 (50.6%); at 2 years n=1853/3308 (56%).	At 6 months, 78% sick listed; at 1 year, 47% sick listed; at 2 years, 20% sick listed; at 3 years, 8% sick listed.			
181	Borchgrevink (1996)	115 worked full-time before RTC. n=78/115 returned to full time work; n=8/115 to part time work; n=1/115	73% did not register for sick leave; 14% sick leave < 14 days immediately after the accident; 8% sick leave > 14			

		on sick leave; n=7/115 on rehab benefits; and n=9/115 received permanent disability pension.<br><br>23 worked part time before RTC. n=8/23 still worked part time; n=6/23 changed to full time; n=3/23 received permanent disability pension.	days; 5% no sick leave immediately after the accident but in follow up period.			
196	Brison & Pickett (2000)		Of persons working for income, 62% missed <1 week of work (mean 6.6 days).	At 6 months, 36% continued to modify their work activities.		
205	Buitenhuis (2009)	1-month post RTC: no work, n=119/728 (16.3%); paid employment n=578/728 (79.4%). Mean (SD) working hours of 32.4 hrs (12.0).<br>6-months post RTC: no work, n=80/448 (17.9%); paid employment n=346/448 (77.2%). Mean (SD) working hours of 32.2 hrs (12.1).<br>12-months post RTC: no work, n=70/384 (18.2%); paid employment n=292/384 (76.0%). Mean (SD) working hours of 32.1 hrs (12.3).	People on work disability 1-month n=247 (33.7%); 6 months n=138 (18.9%); 12 months n=92 (12.6%).			
206	Bunketorp & Carlsson (2002)		WAD group: 34% on sick leave, partial or full disablement pension/sickness benefit, or on retirement pension preceded by a disablement pension.<br>Non-WAD group: 6% partly or fully work disabled due to reasons other than WAD.		n=18/25 was assigned a medical disability because of injuries- ranged from 5-30%, mean of 14%.	
214	Bylund (1998)		Within 2.5 years of RTC 40% of those injured had been on sick leave totalling 12,500 days.<br>Rear-end collisions group: n=15 (15%) males with total 2080 sick leave days; n=28 (28%) females with total 5953 sick leave days. Other crash mechanisms n=33 (21.3%) males with total 2599 sick leave days; n=27 (17.4%) females with total 1883 sick leave days.			
226	Carroll (2012)		Participants off work at 6 weeks n=685/3830; 3 months n=388/3697; 6 months n=237/3343.			

233	Casey (2011)			Out of 180 employed, 38% reported to be unable to continue in their preinjury work capacity.		
234	Casey (2015)			Work disability at 3 months 38%; at 12 months 43%. *In this study work disability was self-reported and defined as those who were unable to work at all, those who had to change jobs as a result of their injury and those who returned to work in a reduced capacity as compared to their usual pre-injury*		
329	de Rome (2012)		Time off work since crash, at 6 months for all participants mean 13.5 (SD 29.8; median 3.0; range 0.0–13.0). For unprotected riders mean = 22.0 (40.3) days, median (IQR) = 7.0 (0.0-14.0) days; partial protection riders mean (SD) = 13.8 (29.6) days, median (IQR) = 2.0 (0.0-15.0) days; and full protection riders mean (SD) = 9.5 (24.9) days, median (IQR) 3.0 (0.0-10.0) days.			
351	Dufton (2012)		At discharge, people off work because of collision acute 56.9% (n=1749/3075), early chronic 52.8% (n= 506/958), chronic 32.7% (n=506/1548).	People modified work duties acute 35.6%, early chronic 38.2, chronic 27.2%.		
352	Dufton (2006)	RTW n=1019 (46.6%) at mean time 31.7 days.				
381	Ettlin (1992)	% not having employment disability, thus (partially) returned to work.<br>3 months n=12; n=3 partial employment disability.<br>1 year n=16; n=5 partial employment disability.<br>2 years n=16; n=5 partial employment disability.<br><br>Full employment disability thus not returned to work: 3 months n = 7; 1- and 2-years n = 0.				
432	Geldman (2008)	For low, medium and higher fitness, RTW rates were at 3 months respectively 31%, 55%, and 53% and at 6 months 69%, 79% and 87%.				

		Across all fitness levels, RTW rates were at 3 months 45/90 = 50% and at 6 months 71/89 = 79.8%.				
440	Gopinath (2015)	RTW at 12 months n=145/170 (85.3%); 24 months n=121/147 (82.3%).		Resumed full duties at 12 months n=119/145 (82.1%); 24 months n=108/121 (89.3%).		
443	Gopinath (2017)	At 24 months, 82% RTW (who worked prior). Just less than 1 in 2 claimants did not report sustained RTW (i.e. were not consistently working throughout the 24 months).		At 24 months, 89% resumed full duties.		
450	Gray (2018)	Failed RTW n=3913/24311 (16%); sustained RTW n=20398/24311 (84%).				
B01	Gray (2018)	Within the 3 years, n=2,199 of those with musculoskeletal injuries (e.g., mild acquired brain injuries excluded) attempted a Gradual RTW (GRTW) pathway at some point, whereas n=26,608 attempted other RTW pathways.				
457	Guest (2017)					No economic loss claim: MSK injuries n=3101/5734 (54.1%); MSK injuries + psych distress n=329/607 (54.2%).
460	Gun (2005)	At 1 year, n=121/135 (90%) RTW.				
498	Herrström & Högstedt (2000)		At 12months, n=40/125 (32%) reported to have used sick leave. n=15/125 sick leave exceeded 4 weeks, n=7/125 (5.6%) remained after 40-52 weeks. Duration of sick leave men (n=51): 2 (1-52) weeks, women (n=74): 3 (1-42) weeks.			
501	Hildingsson (1990)		At follow up (~25 months), n=13/93 reported sick leave.	n=17 had changed jobs, or worked part time, or were looking for other jobs; n=10 were retraining.		
506	Holm (1999)	Year 1989: 63% WAD returned to full work capacity. Year 1994: 69% WAD returned to full work capacity.		Year 1989: partial work disability n=26 (14%); full work disability n = 42 (23%). Year 1994: partial work disability n = 55 (12%); full work disability n = 93 (19%).		
521	Hours (2014)	10% did not RTW at 1 year.	Non-WAD: 47.3% sickness leave (n = 98), median (IQR) 10 (3, 21); Grade 1 WAD: 51.6% sickness leave (n = 32), median (IQR) 14 (4, 21);			

			Grade 2 WAD: 63.3% sickness leave (n = 69), median (IQR) 18 (8, 45).			
522	Hoving (2003)				Neck Disability Index – Work Item mean (SD) 2.2 (1.3)/5; The Northwick Park Neck Pain Questionnaire - Work/housework mean(SD)=1.7(1.2); problem elicitation technique (PET) - work for wages n=37 (52.1%).	
597	Kasch & Jensen (2001)	n = 11/141 (7.8%) did not RTW at 1 year.		4% had returned only to modified job functions at 1 year.		
603	Kasch (2011)	n = 126/138 (91%) RTW after 1 year.				
B02	Kasch (2019)			At 1 year, n=12/143 (8%) did not regain the same work capacity or worked the same amount of hours as before the injury.		
622	Kinzel (2006)	n = 4 RTW; n = 7 did not RTW.				
643	Krogh (2018)			At 1 year, n=15/83 (18%) were non-recovered (defined as not returned to pre-injury work *capacity*).		
687	Leth-Petersen (2009)					Lost earnings capacity (number of patients (proportion)) of 0%: men 204 (0.57), women 431 (0.51), total 635 (0.53);<br>15%: men 60 (0.17), women 201 (0.24), total 261 (0.22);<br>18%: women 1 (0), total 1 (0);<br>20%: men 18 (0.05), women 28 (0.03), total 46 (0.04);<br>25%: men 73 (0.2), women 75 (0.21), total 248 (0.21);<br>30%: men 4 (0.01), women 8 (0.09), total 12 (0.01).
727	Mankovsky-Arnold (2017)	n=28 (27%) RTW (employed full or part-time); n=77 (73%) were work-disabled.				
756	Miettinen (2004)		At 1 year: n=71 (39%) had been on sick leave. Of these, n=23 (12.6%) sick leave < 1week; n=27 (14.58%) 1 week-1 month; n=21 (11.5%) > 1 month (35-365 days).			
757	Miettinen (2002)		At 1 year: 35.3% had been on sick leave. n=21 (10.4%) > 1 month. Of these, n=6 (3%) > 3 months; n=3 > 6 months.			
788	Munjin (2011)		Leave of absence average 104.3 days (median 94, range 24 to 382 days).			

798	Myrtveit (2015)				Reduced work capability was reported by 15.1% (n = 98).	
B03	Nguyen (2019)	At 6 months, 91.0% of the low-risk injury group and 54.6% of the high-risk injury group RTW.				
848	O'Hara (2018)	At 2 years, 63% RTW in an average of 1.01 years (95%CI 0.82-1.20).				At 2 years, monthly income was 62% less than preinjury monthly earnings (mean difference, 117.50 USD; 95%CI 34 to 201 USD). At 2 years, participants accumulated 1.069 USD in debts (95%CI 673 to 1466 USD; 45% of the mean preinjury annual income).
906	Pieske (2010)	At 1 month, n= 8/81 (9.9%) unable to work. At 6 months n=3/81 unable to work (3.7%).	Duration of inability to work in total at 1 month 8/81 (9.9%), at 3 months 5/81 (6.2%), at 6 months 3/81 (3.7%). Junior doctors at 1 month n=5 (14.3%); 3 months n=3 (8.6%); 6 months n=2 (5.7%). More experienced doctors at 1 month n=3 (6.5%); 3 months n=2 (4.3%); 6 months n=1 (2.2%).			
918	Prang (2015)	n = 955 (74%) RTW.				
940	Ratzon (2015)				Degree of disability based on medical file and functional capacity evaluation: WAD group 2/76 had 20% disability, 10/76 had 10% disability, 14/76 had <10% disability, and 50/76 had no disability.<br>Discrepancy between text and figure for reporting of disability in the hip group.	
946	Rebbeck (2006)		The mean number of days taken off from work due to WAD injury median (IQR) for 3, 6 and 12 months is 0.0 (0.0-0.0). Participants that took workdays off at 3 months n=61 (24.4%); 6 months n=30 (20.2%); 2 years n=24 (16.3%).			
971	Rosenthal (1979)	n=0 who worked in a standing occupation returned to work. 50% who were working before injury did not RTW/ required disability assistance.				
991	Sarrami (2016)	At 2 years post-surgery, RTW rate was 37%.		At 2 years post-surgery, return to pre-injury duties was 23%.		

1009	Schreiber (2009)	81.6% were employed before RTC, 25.8% of which stopped working since the accident.				n=13/38 reported decreased work function secondary to RTC.	
1016	Scuderi (2005)	n=12/58 were unable to RTW in any capacity.	Lost days of work. Workers compensation group, at 3 months 2262 days, mean 37.1 days per person (n=61); personal injury group 1093 days, mean 4.7 days per person (n=235).<br>At point of maximum medical improvement, or 2y follow up, total lost days of work: workers compensation group 7107, mean 131.6 days per person; personal injury group 6206 days, mean 28.7 days per person.				
1047	Smed (1997)		At 1m post RTC, n=10/26 no sick leave, n=10/26 max of 7 days leave, n=4/26 still on sick leave.				
1091	Swartzman (1996)	Employment status (2=part time with restrictions and 3= part=time) litigants average 2.4; post litigants 2.7. Hours per week worked outside the home, litigants mean 22.7; post litigants mean 26.4 (not significantly different).					
1163	Virani (2001)		36% took time off work as result of RTC injury symptoms.				
1168	Vos (2008)		At baseline, 36% reported to have taken sick leave.				

Abbreviations: IQR, interquartile range; MSK, musculoskeletal complaints; RTC, road traffic crash; RTW, return to work; WAD, whiplash associated disorder.

Supplementary Material 3

Table 1. Pooled return to work (RTW) percentages by time point and quality of evidence.

ID	Study	Percentage of patients that RTW	95% Confidence Interval
	**1 month**		
54	Ackland (2013)	57.41%	49.41, 65.13
205	Buitenhuis (2009)	79.40%	76.27, 82.28
352	Dufton (2006)	46.64%	44.53, 48.75
906	Pieske (2010)	90.12%	81.46, 95.64
	Random pooled effect size	*69.50% (df=3; n=3156; I^2 99.05%; 95%CI 98.59, 99.36; p<0.001)*	*47.15, 87.83*
	**3 months**		
54	Ackland (2013)	79.63%	72.60, 85.54
381	Ettlin (1992)	71.43%	47.83, 88.72
432	Geldman (2008)	50.00%	39.27, 60.73
	Random pooled effect size	*67.11% (df=2; n=273; I^2 91.37%; 95%CI 77.77, 96.65; p<0.001)*	*44.69, 86.04*
	**6 months**		
162	Biering-Sorensen (2014)	41.96%	40.27, 43.66
205	Buitenhuis (2009)	77.23%	73.06, 81.04
432	Geldman & Cheek (2008)	79.78%	69.93, 87.55
906	Pieske (2010)	96.30%	89.56, 99.23
	Random pooled effect size	*75.87% (df=3; n=3926; I^2 99.20; 95%CI 98.84, 99.45; p<0.001)*	*48.25, 95.06*
	**12 months**		
54	Ackland (2013)	89.51%	83.73, 93.77
162	Biering-Sorensen (2014)	50.56%	48.86, 52.29
205	Buitenhuis (2009)	76.04%	71.45, 80.23
381	Ettlin (1992)	100%	83.89, 100.00
440	Gopinath (2015)	85.29%	79.06, 90.25
460	Gun (2005)	89.63%	83.21, 94.21
521	Hours (2014)	90.14%	80.74, 95.94
597	Kasch & Jensen (2001)	92.20%	86.47, 96.04
603	Kasch (2011)	91.30%	85.30, 95.43
622	Kinzel (2006)	36.36%	10.93, 69.21
	Random pooled effect size	*83.05% (df=9; n=4541; I^2 98.51%; 95%CI 98.05, 98.86; p<0.001)*	*68.62, 93.68*
	**24 months**		
162	Biering-Sorensen (2014)	56.02%	54.30, 57.72
381	Ettlin (1992)	100.00%	83.89, 100.00
440	Gopinath (2015)	82.31%	75.17, 88.11
848	O'Hara (2018)	62.96%	48.74, 75.71
991	Sarrami (2016)	37.35%	26.97, 48.66
	Random pooled effect size	*69.57% (df=4; n=3613; I^2 95.71%; 95%CI 92.49, 97.55; p<0.001)*	*52.12, 84.52*

Supplementary Material 4

Table 1. Pooled sick leave percentages, reported as duration of sick leave or used sick leave at a certain time point.

ID	Study	Duration of reported sick leave (e.g. percentage of participants that reported taking sick leave of the specified duration)	95% Confidence Interval
	**<2 weeks**		
121	Barbosa, 2013	20.00%	14.19, 26.93
181	Borchgrevink, 1996	13.91%	8.17, 21.61
196	Brison & Pickett, 2000	62.04%	56.75, 67.12
756	Miettinen, 2004	12.64%	8.18, 18.36
1047	Smed, 1997	38.46%	20.23, 59.43
	Random pooled effect size	*28.10% (df=4; n=841; I^2 98.05%; 95% CI 96.97, 98.74; p<0.001)*	*8.89 to 52.93*
	**>2 weeks, <1 month**		
121	Barbosa, 2013	10.91%	6.60, 16.69
181	Borchgrevink, 1996	7.83%	3.64, 14.34
756	Miettinen, 2004	14.84%	10.01, 20.85
	Random pooled effect size	*11.60% (df=2; n=462; I^2 41.44%; 95%CI 0.00, 82.18; p=0.18)*	*8.05, 15.70*
	**>1 month**		
121	Barbosa, 2013	20.00%	14.19, 26.93
498	Herrström & Högstedt, 2000	12.00%	6.87, 19.02
756	Miettinen, 2004	11.54%	7.29, 17.10
1047	Smed, 1997	15.39%	4.36, 34.87
	Random pooled effect size	*14.79% (df=3; n=498; I^2 46.18%; 95%CI 0.00, 82.10; p=0.13)*	*10.59, 19.55*

ID	Study	Reported sick leave measured at time point (e.g. percentage of participants that reportedly used sick leave at a certain time point)	95% Confidence Interval
	**1 month**		
205	Buitenhuis, 2009	33.93%	30.49, 37.50
226	Carroll, 2012	17.89%	16.68, 19.14
906	Pieske, 2010	9.88%	4.36, 18.54
1047	Smed, 1997	61.54%	40.57, 79.77
	Random pooled effect size	*27.32% (df=3; n=4665; I^2 97.28%; 95%CI 95.25, 98.45; p<0.001)*	*15.58, 40.95*
	**3 months**		
226	Carroll, 2012	10.50%	9.53, 11.53
906	Pieske, 2010	6.17%	2.03, 13.82
946	Rebbeck, 2006	24.40%	19.21, 30.21
	Random pooled effect size	*13.32% (df=2; n=4028; I^2 94.32%; 95%CI 86.79, 97.56; p<0.001)*	*5.50, 23.87*
	**6 months**		
162	Biering-Sorensen, 2014	77.99%	76.54, 79.39
205	Buitenhuis, 2009	18.96%	16.17, 22.00
226	Carroll, 2012	7.09%	6.24, 8.01
906	Pieske, 2010	3.70%	0.77, 10.44
946	Rebbeck, 2006	20.41%	14.21, 27.83
	Random pooled effect size	*22.74% (df 4; n=7607; I^2 99.91%; 95%CI 99.90, 99.93; p<0.001)*	*0.35, 64.73*
	**12 months**		
162	Biering-Sorensen, 2014	47.01%	45.30, 48.73
205	Buitenhuis, 2009	12.64%	10.31, 15.27
498	Herrström & Högstedt, 2000	32.00%	23.94, 40.93
521	Hours, 2014	52.65%	47.48, 57.77
756	Miettinen, 2004	39.01%	31.88, 46.50
	Random pooled effect size	*35.85% (df=4; n=4721; I^2 98.98%; 95%CI 98.55, 99.29; p<0.001)*	*19.57, 54.02*
	**24 months**		
162	Biering-Sorensen, 2014	20.01%	18.66, 21.42
214	Bylund, 1998	40.00%	33.94, 46.30
501	Hildingsson, 1990	13.98%	7.66, 22.72
946	Rebbeck, 2006	16.33%	10.75, 23.31
	Random pooled effect size	*22.34% (df=3; n=3803; I^2 94.17%; 95%CI 88.22, 97.12; p<0.001)*	*13.32, 32.91*

Supplementary Material 5

Table 1. Sensitivity analysis Return To Work (RTW) proportions.

ID	Study	Proportion of patients that RTW	95% Confidence Interval (or other information that was available)
	**1 month**		
54	Ackland (2013)	57.41%	49.41, 65.13
205	Buitenhuis (2009)	79.40%	76.27, 82.28
352	Dufton (2006)	46.64%	44.53, 48.75
906	Pieske (2010)	90.12%	81.46, 95.64
	Random pooled effect size	*69.50% (df=3; n=3156; I^2 99.05; 95%CI 98.59, 99.36; p<0.01)*	*47.15, 87.83*
	**3 months**		
54	Ackland (2013)	79.63%	72.60, 85.54
432	Geldman (2008)	50.00%	39.27, 60.73
	Random pooled effect size	*65.67% (df=1; n=252; I^2 95.68%; 95%CI 87.53, 98.51; p<0.001)*	*35.30. 90.26*
	**6 months**		
162	Biering-Sorensen (2014)	41.96%	40.27, 43.66
205	Buitenhuis (2009)	77.23%	73.06, 81.04
432	Geldman & Cheek (2008)	79.78%	69.93, 87.55
906	Pieske (2010)	96.30%	89.56, 99.23
	Random pooled effect size	*75.87% (df=3; n=3926; I^2 99.20; 95%CI 98.84, 99.45; p<0.001)*	*48.25, 95.06*
	**12 months**		
54	Ackland (2013)	89.51%	83.73, 93.77
162	Biering-Sorensen (2014)	50.56%	48.86, 52.29
205	Buitenhuis (2009)	76.04%	71.45, 80.23
440	Gopinath (2015)	85.29%	79.06, 90.25
460	Gun (2005)	89.63%	83.21, 94.21
521	Hours (2014)	90.14%	80.74, 95.94
597	Kasch & Jensen (2001)	92.20%	86.47, 96.04
603	Kasch (2011)	91.30%	85.30, 95.43
622	Kinzel (2006)	36.36%	10.93, 69.21
	Random pooled effect size	*80.62% (df=8; n=4520; I^2 98.61%; 95%CI 98.17, 98.95; p<0.001)*	*65.11, 92.37*
	**24 months**		
162	Biering-Sorensen (2014)	56.02%	54.30, 57.72
440	Gopinath (2015)	82.31%	75.17, 88.11
848	O'Hara (2018)	62.96%	48.74, 75.71
	Random pooled effect size	*67.47% (df=2; n=3509; I^2 95.81%; 95%CI 90.92, 98.07; p<0.001)*	*47.75 to 84.40*

Table 2. Sensitivity analysis adjusting for poor quality rated papers. Significance is set at p=0.05.

**Outcome**	**Pooled percentage all studies**	**Pooled percentage good and moderate papers only**	**Z-score**	**Significance**
Reported Return To Work				
1 month	69.50%	69.50%	0	1.00
3 months	67.11%	65.67%	0.35	0.73
6 months	75.87%	75.87%	0	1.00
12 months	83.05%	80.62%	3.00	<0.01*
24 months	69.57%	67.47%	1.91	0.06

Supplementary Material 6

Table 1. Sick leave proportions good and moderate quality studies only.

**ID**	**Study**	**Proportion of patients that reported *duration* of sick leave**	**95% Confidence Interval**
	**<2 weeks**		
181	Borchgrevink, 1996	13.91%	8.17, 21.61
756	Miettinen, 2004	12.64%	8.18 to 18.36
	Random pooled effect size	*13.37% (df=1; n=297; I^2 0.00%; 95%CI 0.00, 0.00; p=0.73)*	*9.76 to 17.45*
	**>2 weeks, <1 month**		
181	Borchgrevink, 1996	7.83%	3.64 to 14.34
756	Miettinen, 2004	14.84%	10.01 to 20.85
	Random pooled effect size	*11.61% (df=1; n=297; I^2 69.77%; 95%CI 0.00, 93.20; p=0.07)*	*5.75, 19.18*
	**>1 month**		
498	Herrström & Högstedt, 2000	12.00%	6.87, 19.02
756	Miettinen, 2004	11.54%	7.29, 17.10
	Random pooled effect size	*11.97% (df=1; n=307; I^2 0.00%; 95%CI 0.00, 0.00; p=0.88)*	*8.60, 15.82*
**ID**	**Study**	**Proportion of patients that reported sick leave *by time point***	**95% Confidence Interval**
	**1 month**		
205	Buitenhuis, 2009	33.93%	30.49, 37.50
226	Carroll, 2012	17.89%	16.68, 19.14
906	Pieske, 2010	9.88%	4.36, 18.54
	Random pooled effect size	*20.40% (df=2; n=4639; I^2 97.78%; 95%CI 95.81, 98.83; p<0.001)*	*9.90, 33.49*
	**3 months**		
226	Carroll, 2012	10.50%	9.53, 11.53
906	Pieske, 2010	6.17%	2.03, 13.82
	Random pooled effect size	*9.76% (df=1; n=3778; I^2 33.34%; 95%CI 0.00, 0.00; p=0.22)*	*6.91, 13.03*
	**6 months**		
162	Biering-Sorensen, 2014	77.99%	76.54, 79.39
205	Buitenhuis, 2009	18.96%	16.17, 22.00
226	Carroll, 2012	7.09%	6.24, 8.01
906	Pieske, 2010	3.70%	0.77, 10.44
	Random pooled effect size	*23.28% (df=3; n=7460; I^2 99.93%; 95%CI 99.92, 99.94; p<0.001)*	*0.00, 71.09*
	**12 months**		
162	Biering-Sorensen, 2014	47.01%	45.30, 48.73
205	Buitenhuis, 2009	12.64%	10.31, 15.27
498	Herrström & Högstedt, 2000	32.00%	23.94, 40.93
521	Hours, 2014	52.65%	47.48, 57.77
756	Miettinen, 2004	39.01%	31.88, 46.50
	Random pooled effect size	*35.85% (df=4; n=4721; I^2 98.98%; 95%CI 98.55, 99.29; p<0.001)*	*19.57, 54.02*
	**24 months**		
162	Biering-Sorensen, 2014	20.01%	18.66. 21.42
	Random pooled effect size	-	-

Table 2. Sensitivity analysis adjusting for poor quality rated papers. Significance is set at p=0.05.

Outcome	Pooled percentage all studies	Pooled percentage good and moderate papers only	Z-score	Significance
Sick leave duration				
<2 weeks	28.10%	13.37%	5.09	<0.001*
>2 weeks, <1 month	11.60%	11.61%	-0.004	1.00
>1 month	14.79%	11.97%	1.13	0.26
Reported sick leave by time point				
1 month	27.32%	20.40%	7.83	<0.001*
3 months	13.32%	9.76%	4.91	<0.001*
6 months	22.74%	23.28%	-0.79	0.43
12 months	35.85%	35.85%	0	1.00
24 months[a]	22.34%	-	-	-

[a] Three out of four studies were rated as poor quality studies, therefore sensitivity analysis could not be performed.

Supplementary Material 7

Table 1. Factors associated with work-related outcomes.

ID		Work Outcomes				
		**RTW**	**Sick leave**	**Work capacity**	**Work ability**	**Productivity loss**
54	Ackland (2013)	At any given time during the 12-month follow-up period, patients with minor limb compared to other injuries were 55% less likely to have returned to work/normal daily activities (Adjusted HR = 0.45, 95% CI 0.31-0.89, p<0.01), patients with HADS depression sub-scores of 11 or more at outpatient assessment had a 57% lower probability of having returned to work than patients with sub-scores of less than 11 (Adjusted HR 0.43, 95% CI 0.23-0.79, p<0.01), and patients with high annual earning capacity were 2.34 times more likely to have returned to work (Adjusted HR 2.34, 95% CI 1.44-3.78, p<0.01).				
121	Barbosa (2014)		No statistical significant association between time occurrence of facial injury and time off work (p=0.053).			Statistically significant association between occurrence of facial injury and absenteeism. (p=0.024).
151	Berecki-Gisolf (2013)		Time off work after 6 months: female gender, older age, opioid prescription, dislocations (only if 1-7 days in hospital) vs. sprains/strains, labourers vs. managers/administrators or professionals (only if 1-7 days in hospital).			
181	Borchgrevink (1996)	Significantly more women than men had claimed rehab or permanent disability pension (Chi=4.3, p<0.05).				
205	Buitenhuis (2009)		No significant difference self-employed or paid employment regarding work disability at 1, 6, 12 months.<br><br>Work disability at 1 month was independently associated with higher neck pain intensity, more severe restriction of neck movements, more			

			intense concentration complaints, and consumption of medication at 1 month. Work disability at 6 months was independently associated with concurrent neck pain intensity, concentration complaints at 6 months and relatively intense concentration complaints at 1 month. Work disability at 12 months was independently associated with concurrent complaints at 12 months, higher age and more concentration complaints.			
206	Bunketorp & Carlsson (2002)				Women with WAD were more likely than men to be work disabled, but the difference was not statistically significant (P=0.115).	
214	Bylund (1998)		Women had a longer average sick leave than did men, and a higher proportion of them took sick leave. Those involved in rear-end collisions had the longest average sick leave compared to other crash mechanisms.			
234	Casey (2015)			Those who were unable to continue in their pre injury work capacity scored significantly worse on all health outcome measures, SF36-physical ($p<0.001$), SFS6-mental ($p<0.001$) and PCS ($p<0.001$) compared with those who were employed and continued to work unaffected. Predictors of higher disability levels were helplessness ($p<0.001$), being unable to continue in pre-injury work capacity ($p<0.001$) and older age ($p<0.001$).		
351	Dufton (2012)	Patients in the early chronic group were more likely to have returned to work compared to the chronic group ($p < 0.05$).				
440	Gopinath (2015)	Potential risk factors for RTW that were considered: age, sex, marital status, education level, pre-injury paid work status, BMI, pre-injury health variables, hospital admission, ness ISS, pain severity, whiplash,				

		fracture, OMPSQ score, EQ-5D VAS, SF-12 MCS and PCS.<br><br>Multivariable model for RTW at 12m: significance for OMPSQ <50 at baseline, SF-12 MCS (9% greater likelihood) at baseline; non-significance for age and gender.<br>Multivariable model for RTW at 24m: significance for not having pre-injury chronic illness, not having hospital admission, SF-12 MCS; non-significance for age and gender.<br>Multivariable model for sustained RTW at both 12 and 24m: significance for not admitted to hospital, SF-12 MCS, non-significance for age and gender, overweight/obese.<br>Multivariable model for full duties at 12m: significance for younger age, OMPSQ without disability scores, all others non-significant.<br>Multivariable model for full duties at 24m: significance for younger age, SF-12 PCS, all others non-significant.				
443	Gopinath (2017)	A range of psychosocial factors were the strongest predictors of RTW in multivariable regression models.				
450	Gray (2018)	Significant covariates on the odds of having at least one failed RTW were sex, age, injury type, socioeconomic group, severity group.				
B01	Gray (2018)	Least likely to attempt gradual RTW were males, individuals with contusions, abrasions, sprains, strains, non-limb fractures and those from the most advantaged socioeconomic group. Those admitted to hospital were 88% more likely to relapse.<br><br>Of those that followed a gradual RTW pathway, those aged 15–24 years were most likely to succeed. Those with whiplash, internal injuries and those admitted to hospital				

		were least likely to succeed.				
460	Gun (2005)	Consulting a lawyer was associated with a 5-fold lesser chance of returning to work at 1 year. Adjusting for baseline scores removed the significance of this relationship.				
501	Hildingsson (1990)		Positive correlation between duration of symptoms and sick leave (r=0.76, p=0.001). No significant correlation between previous chronic headache, neck complaints or professional belonging and sick leave.			
506	Holm (1999)			In all cases, those with partial or full work disability were older (over 40 years), had greater medical impairment (over 15%), and lower professional status than those with no work disability.		
521	Hours (2014)	WAD grades did not differ significantly for RTW at 1 year.	Grade 2 casualties had longer sick leave (p = 0.05).			
522	Hoving (2003)				Neck Disability Index – Work Item and The Northwick Park Neck Pain Questionnaire - Work/housework Item were correlated with the overall problem elicitation technique (PET) – work for wages score (r=0.53, r=0.59 respectively)	
687	Leth-Petersen (2009)					Individuals having been awarded a lost earnings capacity (LEC) less than 15% do not experience a significant reduction in the employment propensity. A LEC of 15–30% exhibits a reduced employment propensity at all horizons (same for both women and men).<br>Only the more severely injured persons, those with assessed earnings loss in the interval 15–30% experience a significant loss in earnings at all horizons. Men are constant but women are not (dramatic reduction 1–3 years, slight recovery 4–5 years). For persons with a smaller assessed earnings loss, not entitling them for compensation, there does not

						appear to be any significant reduction in earnings at any horizon.
727	Mankovsky-Arnold (2017)	Multiple regression revealed index of multi-site pain, index of sensitivity to movement-evoked pain significantly contributed to employment status.				
756	Miettinen (2004)		Women had significantly longer sick leave than men (p<0.05). The single persons managed better in respect the length of the sick leave than those who were either married, divorced or widowed (p<0.05). Age, education, the speed of the vehicle, subjects' position in the car or the use of the seatbelt had no correlation to the length of sick leave.			
798	Myrtveit (2015)				Reduced working capability was most strongly associated with preferring to take medications (OR=3.53; 95% CI 2.13 to 5.86), sickness absence (OR=3.05; 95% CI 1.80 to 5.17) and being referred to a physiotherapist/ chiropractor (OR=3.03; 95% CI 1.33 to 6.91). Keep living as usual was protective for reduced work capability (OR=0.09; 95% CI 0.01 to 0.64). Participants who believed that a change of lifestyle could make them better were protected against reduced work capability at 12 months (OR=0.11; 95% CI 0.01 to 0.78).	
B03	Nguyen (2019)	At 6 months, 91.0% of the low-risk injury group and 54.6% of the high-risk injury group RTW.<br><br>The adjusted risk ratios of returning to work fully were statistically significant in neck (RR = 1.68, 95% CI: 1.09 to 2.59) and lower back (RR = 2.79, 95% CI: 1.17 to 6.68) injury groups.				
848	O'Hara (2018)					At 2 years, no association between surgical treatment received and monthly income, debt, employment or dependents.

906	Pieske (2010)	Strong correlation between incidences of neck pain NRS>2, analgesic medication, and work-off during study (p<0.01).				
918	Prang (2015)	Women receiving support from family, regardless of the amount, had decreased odds for RTW, whereas 'definite' support from friends increased the odds of RTW. No such differences were observed among men. For both men and women, support from employers was positively associated with RTW. Younger persons, persons with a university level education were more likely to have RTW. Persons who were widowed, separated, or divorced were less likely to have RTW. Participants living in Melbourne (p = .03), employed as professionals (p < .001), earning more than $50,000 (p = .003), sustained a dislocation (p = .001) were more likely to have RTW.				
991	Sarrami (2016)			No significant associations between the potential predictors (age, gender, surgery type, surgery location, Socio-Economic Indexes for Areas (SEIFA)) and work capacity.		
1016	Scuderi (2005)		Participants in the workers compensation group lost significantly more work days than participants in the personal injury group.			
1091	Swartzman (1996)	No difference in hours per week worked outside the home between current litigants and post litigants. Adjusted for months since pain began, initial number of body sites affected, initial number of poor prognostic body sites affected.				
1168	Vos (2008)		Sick leave percentage higher in MVA group than in remaining cohort (p=0.037).			
